# Proinflammatory chemokine CXCL14 activates MAS-related G protein-coupled receptor MRGPRX2 and its putative mouse ortholog MRGPRB2

**DOI:** 10.1038/s42003-023-05739-5

**Published:** 2024-01-06

**Authors:** Ghazl Al Hamwi, Vigneshwaran Namasivayam, Beatriz Büschbell, Robin Gedschold, Stefan Golz, Christa E. Müller

**Affiliations:** 1 PharmaCenter Bonn, Pharmaceutical Institute, Pharmaceutical & Medicinal Chemistry, University of Bonn, An der Immenburg 4, 53121 Bonn, Germany; 2 Lead Identification & Characterization, Pharma Research and Development Center, Bayer AG, Wuppertal, Germany

**Keywords:** Receptor pharmacology, Molecular medicine

## Abstract

Patients with idiopathic pulmonary fibrosis show a strongly upregulated expression of chemokine CXCL14, whose target is still unknown. Screening of CXCL14 in a panel of human G protein-coupled receptors (GPCRs) revealed its potent and selective activation of the orphan MAS-related GPCR X2 (MRGPRX2). This receptor is expressed on mast cells and − like CXCL14 − upregulated in bronchial inflammation. CXCL14 induces robust activation of MRGPRX2 and its putative mouse ortholog MRGPRB2 in G protein-dependent and *β*-arrestin recruitment assays that is blocked by a selective MRGPRX2/B2 antagonist. Truncation combined with mutagenesis and computational studies identified the pharmacophoric sequence of CXCL14 and its presumed interaction with the receptor. Intriguingly, C-terminal domain sequences of CXCL14 consisting of 4 to 11 amino acids display similar or increased potency and efficacy compared to the full CXCL14 sequence (77 amino acids). These results provide a rational basis for the future development of potential idiopathic pulmonary fibrosis therapies.

## Abstract (web summary)

This study reveals that the proinflammatory chemokine CXCL14 activates MAS-related G protein-coupled receptor MRGPRX2; these results provide a rational basis for the future development of idiopathic pulmonary fibrosis therapies.

## Introduction

Idiopathic pulmonary fibrosis (IPF) is a fatal interstitial lung disease of unknown cause^1,2^. It is characterized by irreversible scarring of the tissue surrounding the air sacs in the lungs, leading to death due to respiratory failure^1^. The prevalence of IPF is estimated to be around 10-60 per 100,000 people worldwide, making it the most common type of fibrotic lung diseases^1,2^. Nearly half of the IPF patients die within 2–3 years after diagnosis^2,3^. Fibrosis is the result of exaggerated tissue repair, which is controlled by the innate and adaptive immune system. Chemokines, cytokines promoting chemokinesis, are a family of small, structurally related proteins that play an essential role in immune functions^4^. They are involved in the mobilization and trafficking of various leukocytes and mesenchymal progenitor cells such as fibrocytes^5^. The chemokine family of bioactive peptides in humans consists of 47 members that are classified into four main subfamilies based on the sequential positioning of the first two cysteine (C) residues (where X is any amino acid): CXC, CC, CX3C, and C^6^. Most chemokines interact with one or several of the 23 chemokine receptors, that are found on the surface of their target cells^6^. Chemokine receptors belong to the *γ*-branch of class A, rhodopsin-like G protein-coupled receptors (GPCRs). They can be divided into conventional receptors with G protein-mediated downstream signaling, and atypical receptors that are not coupled to G proteins^5,7^. Each chemokine receptor is paired with at least one chemokine, however, two chemokines, CXCL14 and CXCL17, remain without receptor and are still considered orphan. The *CXCL14* gene is located on chromosome 5 in humans and encodes for a 99-amino acid precursor protein, which is processed to a mature 77-amino acid protein with a molecular weight of 9.4 kDa, also known as BRAK (breast and kidney-expressed chemokine)^8^. A variety of immune cells as well as nonimmune cells secrete CXCL14 either constitutively or after activation. Immune cells such as monocyte-derived immature dendritic cells and skin mast cells (MCs) secrete CXCL14 under basal conditions^9,10^. Monocytes and B cells isolated from human peripheral blood express CXCL14 after stimulation with lipopolysaccharide^11^. Several types of immune cells that do not express CXCL14 exhibit a chemotactic response towards the chemokine^12^. These include CD56^+^ natural killer (NK) cells and neutrophils, while naïve or activated T cells are non-responsive^8,11,13,14^. Non-immune cells, for example, skin keratinocytes, dermal endothelial cells, and dermal fibroblasts (co-localized with macrophages) can also secrete CXCL14^12,15^.

Microarray RNA expression analysis revealed that *CXCL14* is only expressed at relatively low concentrations in normal healthy airway epithelium^16^. However, CXCL14 levels can be massively upregulated in lung disorders, both on the mRNA and the protein level. Lung tissues from a surgical biopsy of IPF patients were shown to overexpress CXCL14 compared to normal lung tissue using real-time quantitative polymerase chain reaction, *immunohistochemistry*, and Western blot^17–19^. High CXCL14 expression is correlated with poor survival of IPF^18^ and lung cancer patients^16^. CXCL14 upregulation was reported in other lung diseases as well, including chronic obstructive pulmonary disease (COPD)^16^, asthma^20^, and pulmonary embolism^21^. Externally induced lung distress, for example, smoking^16^ or bacterial infections^22^ also led to CXCL14 overexpression. Besides lung diseases, CXCL14 has been linked to further inflammatory diseases, such as rheumatoid arthritis^23^, psoriasis^24^, atopic dermatitis^24^, and diabetes^25^. Although reports have linked CXCL14 to cancer, it is unclear whether CXCL14 displays anti-tumor^26,27^ or tumor-promoting properties^27,28^.

The cognate receptor of CXCL14 is still unknown. This chemokine does not appear to directly activate any conventional or atypical chemokine receptor^29^. Several studies suggested that CXCL14 may allosterically modulate the CXCL12/CXCR4 axis^30,31^. Yet, its exact modulatory role, positive or negative^30,31^, and whether it shows an effect at all, is debated^32^. Although it did not induce *β*-arrestin recruitment via the atypical chemokine receptor 2 (AKCR2)^29^, CXCL14 was implicated in breast cancer progression mediated by ACKR2^28,33^. CXCL14 was also found to promote breast cancer cell progression by another orphan GPCR, GPR85^34^.

Since all known chemokine receptors are GPCRs, it is most likely that CXCL14 exerts its effects via a GPCR as well. The elucidation of the target receptor of CXCL14 would greatly facilitate the understanding of its role in health and disease. Moreover, it is a precondition for subsequent target validation and drug development.

In the present study, we discovered that the orphan chemokine CXCL14 potently and selectively activates the MAS-related GPCR MRGPRX2, a GPCR predominantly expressed in mast cells^35,36^, and like CXCL14 overexpressed in lung inflammation^20,37^ and other inflammatory diseases e.g. rheumatoid arthritis^23,38^ and atopic dermatitis^24,39^. Truncation of the peptide and structural analysis led to the identification of its pharmacophore.

## Results

### CXCL14 upregulation in idiopathic pulmonary fibrosis patients

**Fig. 1:**
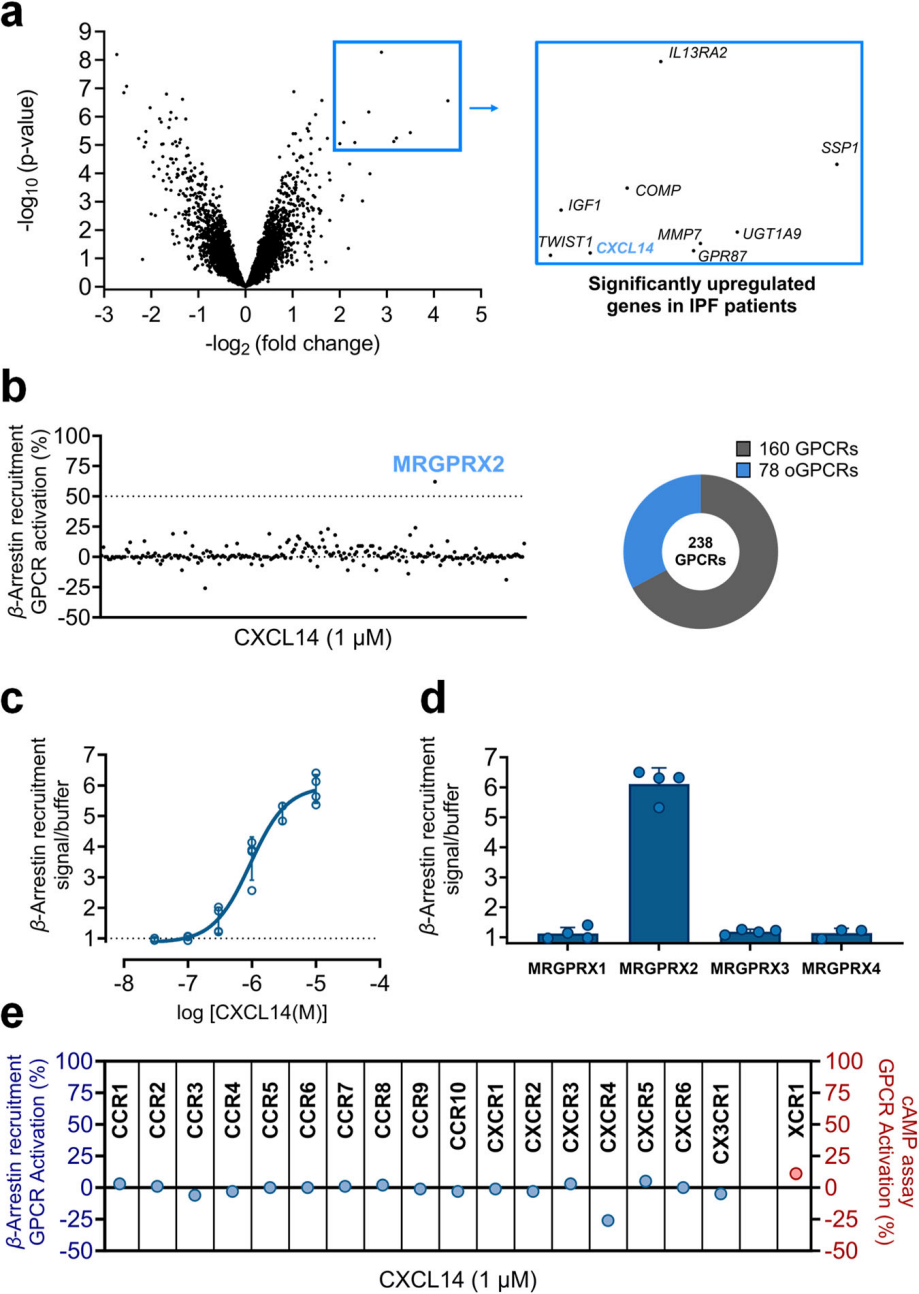
Identification of a target receptor for CXCL14. **a** A volcano plot of gene expression (8912 genes) of healthy (11 lung samples) versus IPF (13 lung samples) groups displaying statistical significance (-log_10_ *p*-value) versus magnitude of change (-log_2_ fold change), using the online Gene Expression Omnibus database (GEO) and the Graphpad Prism software (accession number: GSE2052)^40–43^. Genes in the blue box are significantly upregulated in IPF patients with an adjusted *p*-value cut-off of 0.005 and a more than 4-fold change in expression, see Supplementary Data 1 for more details. Genes coding for insulin-like growth factor 1 (*IGF1*), *CXCL14*, secreted phosphoprotein 1 (*SPP1*), twist family transcription factor 1 (*TWIST1)*, matrix metallopeptidase 7 (*MMP7*), uridine 5’-diphosphoglucuronosyltransferase family 1 member A9 (*UGT1A9*), G protein-coupled receptor 87 (*GPR87*), cartilage oligomeric matrix protein (*COMP*), and interleukin 13 receptor subunit alpha 2 (*IL13RA2*); see Supplementary Data 1 for more details. **b** Screening of GPCRs using the *β*-arrestin recruitment assay: CXCL14 (1 µM) was screened at 160 well-known GPCRs and 78 orphan GPCRs (oGPCRs). For known GPCRs, The effect of CXCL14 was normalized to the maximal effect of the standard agonist of each investigated receptor (for further details see Supplementary Data 2). **c** Concentration-response curves of CXCL14 at MRGPRX2 in *β*-arrestin recruitment assays using *β*-arrestin-Chinese hamster ovary cells (*β*-arrestin-CHO) recombinantly expressing MRGPRX2 (CXCL14, EC_50_ 0.905 ± 0.548 µM), the data are means ± SD of *n* = 4 biological replicates. **d** Effect of CXCL14 (10 µM) in *β*-arrestin recruitment assays at all MRGPRX subtypes (MRGPRX1, MRGPRX2, MRGPRX3, and MRGPRX4), each of which was recombinantly expressed in *β*-arrestin-CHO cells, the data are means ± SD of *n* = 3 or 4 biological replicates. **e** Effect of CXCL14 (1 µM) in *β*-arrestin recruitment assays at all chemokine receptors (CCR1, CCR2, CCR3, CCR4, CCR5, CCR6, CCR7, CCR8, CCR9, CCR10, CXCR1, CXCR2, CXCR3, CXCR4, CXCR5, CXCR6, and CX3CR1). The chemokine receptor XCR1, coupled to G*α*_i_, was tested in cAMP assays. The effect of CXCL14 was normalized to the maximal effect of the standard agonist of each chemokine receptor (for further details see Supplementary Data 2).

With the aim to identify potential drug targets that would enable the development of therapies for IPF, we performed a mRNA expression analysis comprising 8911 genes. Expression in lung tissues of 13 IPF patients was compared with controls from 11 healthy donors utilizing a gene dataset from the Gene Expression Omnibus (accession number: GSE2052)^40–43^. The genes were filtered based on statistical significance to calculate the differences in gene expression between healthy donors and IPF patients. A total of 56 genes was identified to significantly differ in IPF patient as compared to healthy donor samples, considering an adjusted *p*-value of less than 0.005 as a threshold (Fig. 1a, see Supplementary Data 1). Among the hereby identified 56 genes, nine genes displayed at least 4-fold increased expression in IPF patients (Fig. 1a; see Supplementary Data 1 for further information). These upregulated genes can be grouped according to their functional roles. Extracellular matrix remodeling genes included *SPP1* (secreted phosphoprotein 1), *MMP7* (matrix metallopeptidase 7), and *COMP* (cartilage oligomeric matrix protein). Genes encoding growth and transcription factors included *IGF1* (insulin-like growth factor 1) and *TWIST1* (twist family transcription factor 1). Genes involved in immune response and cellular signaling included *CXCL14*, *IL13RA2* (interleukin 13 receptor subunit alpha 2) and GPR87 (lysophosphatidic acid receptor). One identified gene, *UGT1A9* (uridine 5’-diphosphoglucuronosyltransferase family 1 member A9), is involved in metabolism, (see Supplementary Data 1 for further information). Among these genes, *CXCL14* (encoding for the orphan chemokine CXCL14) appeared to be of particular interest due to its reported unique expression pattern, its roles in immune regulation and inflammation, and as a result, its potential clinical relevance and therapeutic potential. However, no receptor that is activated by CXCL14 has so far been reported. As a next step, we therefore set out to identify its target receptor.

### Identification of a target receptor for CXCL14

CXCL14 was screened in a GPCR panel using *β*-arrestin recruitment assays based on enzyme (*β*-galactosidase) complementation^44^. In this system, cells recombinantly express a *β*-arrestin subunit fused to a large fragment of *β*-galactosidase, and a GPCR that is fused to a small fragment of the enzyme. CXCL14 (1 µM) was tested at 160 well-known GPCRs (including the chemokine receptors) and 78 orphan GPCR, whose cognate ligands are still unknown (Fig. 1b; Supplementary Data 2 for more details). The only hit out of the investigated 238 GPCRs was MRGPRX2. The receptor was activated by CXCL14 in a concentration-dependent manner with an EC_50_ value of 0.905 µM (see Fig. 1c). The previously reported MRGPRX2 agonist proadrenomedullin N-terminal peptide-20 (PAMP-20; sequence: ARLDVASEFRKKWNKWALSR) was used as a positive control (see Supplementary Fig. 1a)

As a next step, the selectivity of CXCL14 for MRGPRX2 versus the other MRGPRX subtypes, MRGPRX1, MRGPRX3, and MRPGPRX4, was studied (see Fig. 1d). CXCL14 did not induce any significant *β*-arrestin recruitment when tested in the same assay system at these receptors at a concentration of 10 µM (see Fig. 1d for the effect of CXCL14, and Supplementary Fig. 1b and Supplementary Data 2 for controls of MRGPRX subtypes).

CXCL14 did not activate any other chemokine receptor, however, it appeared to inhibit CXCR4 constituent activity in the *β*-arrestin recruitment assays (see Fig. 1e, Supplementary Fig. 2).

### Validation of CXCL14 as an MRGPRX2 agonist in G protein-dependent assays

**Fig. 2:**
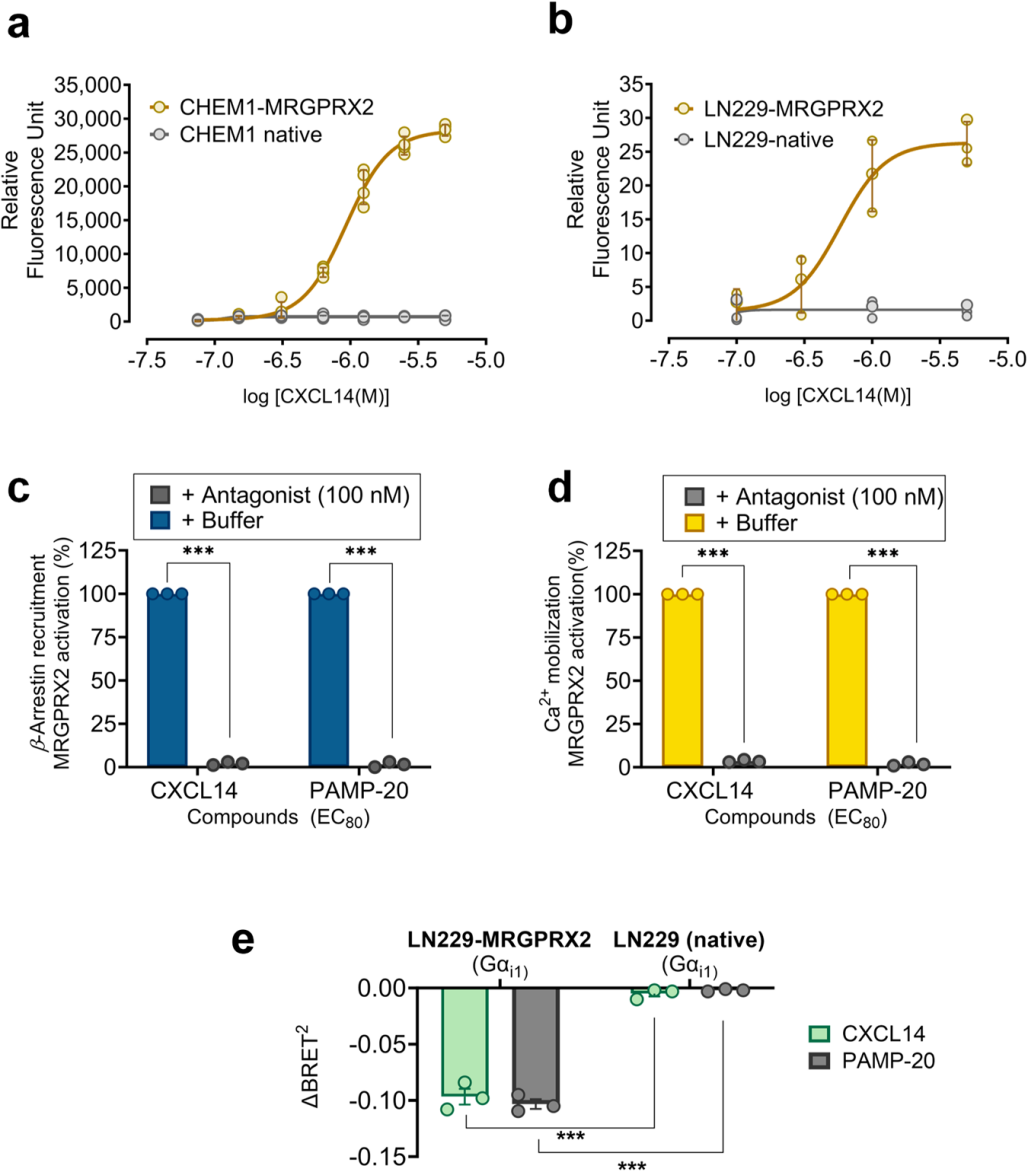
Characterization of CXCL14 as MRGPRX2 agonist. **a** Concentration-response curve of CXCL14 in calcium mobilization assays. An EC_50_ value of 0.889 ± 0.163 µM was determined in CHEM-1 cells recombinantly expressing MRGPRX2, but not the parental CHEM1 cell line; data are means ± SD of 4 biological replicates. **b** Concentration-response curve of CXCL14 at MRGPRX2 in calcium mobilization assays using LN229 cells recombinantly expressing MRGPRX2 (CXCL14, EC_50_ 0.504 ± 0.202 µM); data are means ± SD of 4 biological replicates. Inhibition of calcium mobilization induced by CXCL14 (1 µM) and PAMP-20 (1 µM) by the MRGPRX2 antagonist PSB-172656 (100 nM) (**c**) in calcium mobilization assays using LN229 cells recombinantly expressing MRGPRX2 and **d** in *β*-arrestin recruitment assays using *β*-arrestin-CHO cells recombinantly expressing MRGPRX2. Data represent means ± SD of 3 biological replicates.; ***p < 0.001 multiple t test. **e** G*α*_i1_ protein dissociation due to CXCL14-induced MRGPRX2 activation, determined by a BRET-based assay (TRUPATH). CXCL14 (1 µM) and PAMP-20 (1 µM) were added to LN229 cells recombinantly expressing MRGPRX2 and G*α*_i1_RLuc8, G*β*_3,_ and G*γ*_9_, and to LN229 cells recombinantly expressing only G*α*_i1_RLuc8, G*β*_3,_ and G*γ*_9_, but not MRGPRX2 (data are means ± SD of 3 biological replicates; ***p < 0.001 multiple t-test.

To confirm the initial results obtained in the *β*-arrestin recruitment assays, we established calcium mobilization assays in two different recombinant cell lines, CHEM-1 and LN229 cells, respectively. CHEM-1 cells are adherent rat hematopoietic cells with a high endogenous expression level of the promiscuous G*α*_15_ protein enabling the measuring of calcium signaling of a large variety of GPCRs. CXCL14 was found to activate MRGPRX2 in a concentration-dependent manner showing an EC_50_ value of 0.889 µM in CHEM-1 cells recombinantly expressing MRGPRX2, but not in the parental non-transfected CHEM-1 cell line (see Fig. 2a). The known MRGPRX2 agonist PAMP-20 and was used as a positive control (see Supplementary Fig 3a, b). Subsequently, we employed recombinant human glioblastoma LN229 cells, a human cell line derived from glioblastoma multiforme tumors. These cells express MRGPRXs endogenously and therefore possess the necessary signaling proteins associated with MRGPRXs^39,40^. Indeed, LN229 cells overexpressing MRGPRX2 displayed CXCL14-induced calcium mobilization with an EC_50_ value of 0.504 µM, while non-transfected LN229 cells showed no activation by CXCL14 (see Fig. 2b).

In both, calcium mobilization as well as *β*-arrestin recruitment assays, MRGPRX2 activation was completely blocked by a selective MRGPRX2 antagonist (PSB-172656) (see Figs. 2c and d).

Moreover, CXCL14 (1 µM) induced G*α*_i1_ dissociation in LN229 cells recombinantly expressing MRGPRX2 and the G_i1_ protein complex (G*α*_i1_RLuc8, G*β*_3,_ and G*γ*_9_), but not in cells expressing only the G_i1_ protein complex. This was measured by a bioluminescence resonance energy transfer (BRET) assay (TRUPATH) that allows the direct observation of G*α* protein activation^45^. (see Fig. 2e).

### Identification of the CXCL14 pharmacophore by testing large fragments

**Fig. 3:**
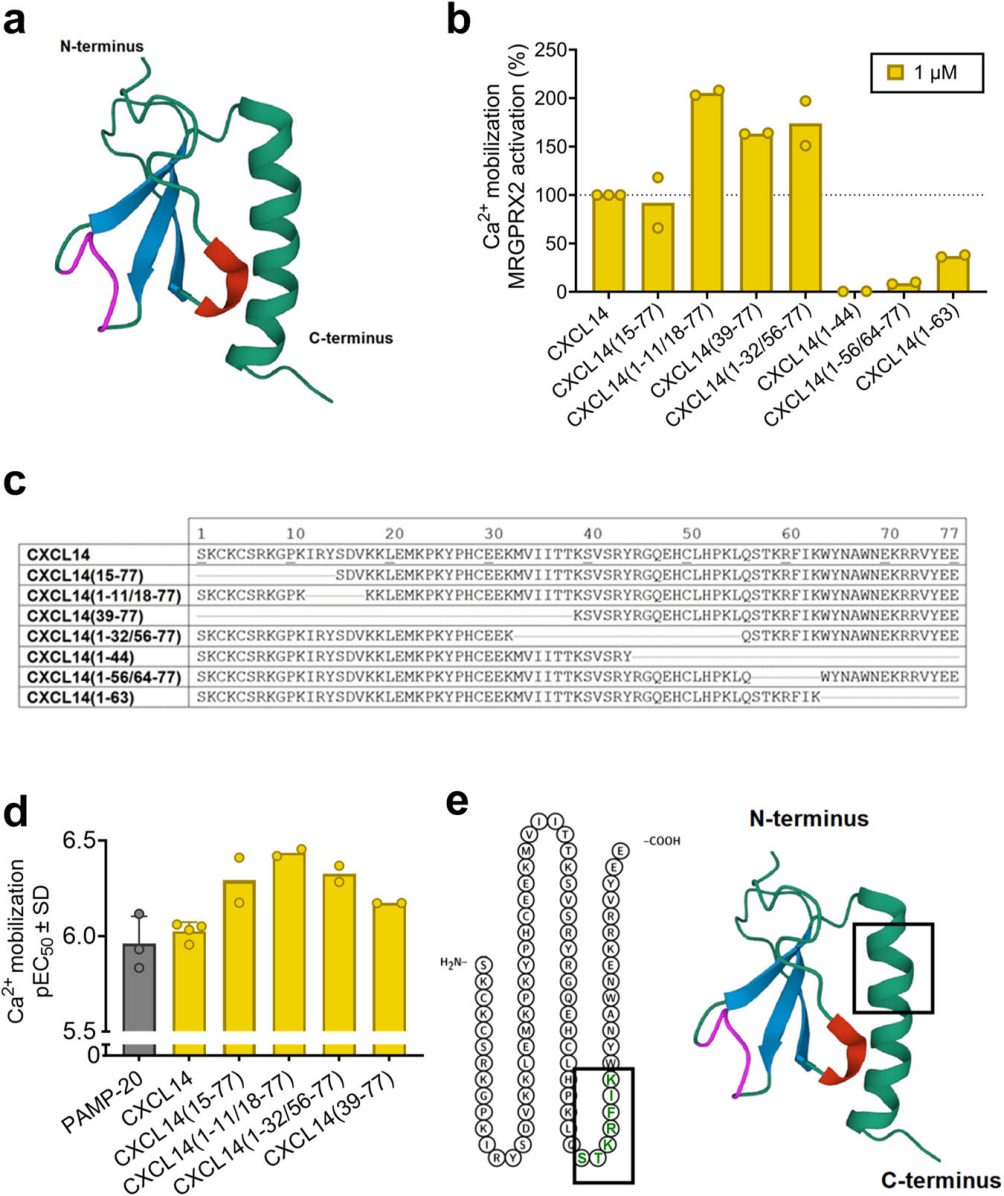
Identification of the pharmacophore of CXCL14 (large fragments). **a** 3D structure of CXCL14 (PDBid: 2HDL) (green, backbone and C-terminal *α*-helix; blue, *β*-strands; red, 3_10_ helix; magenta, unique loop of CXCL14). **b** Effects of large CXCL14 fragments (1 µM) on MRGPRX2 activation measured in calcium mobilization assays using CHEM1 cells recombinantly expressing MRGPRX2, the effect was normalized to 1 µM CXCL14 (100%). **c** Sequences of the investigated CXCL14 fragments. **d** pEC_50_ values of active CXCL14 fragments in comparison to the known MRGPRX2 agonist PAMP-20. Data are means ± SD of 3 to 4 biological replicates, or means of biological duplicates, respectively. Sigmoidal dose-response parameters (variable slope) were used to analyze the data. **e** 2D- and 3D-structure of CXCL14: the 7 amino acid stretch, STKRFIK, that was removed in the largest inactive fragment CXCL14(1-56/64-77), is highlighted by a rectangle.

Human CXCL14 consists of 77 amino acids. CXCL14 adopts the canonical chemokine folding, which consists of an N-terminal loop, three *β*-strands (antiparallel *β*-sheet), and a C-terminal α-helix (see Fig. 3a)^46^. In addition, there are two important loops, the chemokine conserved 3_10_ helix (^14^YSD16) that connects the N-loop to *β*-strand 1 and interacts with the C-terminal *α*-helix through an aromatic anchor (Y14 interacting with F61 and W64), and the unique loop of CXCL14 (^41^VSRYR45) between the *β*2 and *β*3 strands (see Fig. 3a). We investigated various large CXCL14 fragments in calcium mobilization assays using CHEM1 cells overexpressing MRGPRX2 to determine the sequence of CXCL14 that interacts with the receptor (see Figs. 3b and 3c). N-terminal truncation of 14 amino acids in CXCL14 was tolerated, CXCL14(15-77) showing an EC_50_ value of 0.751 µM with a maximal activation similar to that of CXCL14 (see Figs. 3c, d). This indicates that the 3D-fold is not essential for MRGPRX2 activity, as two disulfide bonds were removed upon the N-terminal truncation. The removal of the 3_10_ helix (^14^YSD16) in CXCL14(1-11/18-77) even improved the potency of the peptide by 3-fold compared to CXCL14 (EC_50_ 0.379 µM) and led to a super-agonistic effect (E_max_ 205**%**, normalized to the maximal effect of CXCL14) (see Figs. 3c and d). Since the N-terminal domain did not appear to be required for MRGPRX2 activation, a larger stretch of 33 N-terminal amino acids was removed yielding CXCL14(39-77). This massive N-terminal truncation was similarly well tolerated (EC_50_ 0.680 µM, E_max_ 164%) indicating that the pharmacophore is located either in the central or the C-terminal part of CXCL14 (see Figs. 3c, d). Subsequently, CXCL14(1-32/56-77), lacking 24 amino acids from the central part, was tested and found to activate MRGPRX2 equipotently to CXCL14(39-77) (EC_50_ 0.678 µM, E_max_ 175%) (see Figs. 3c, d). Next, CXCL14(1-44), CXCL14(1-56/64-77) and CXCL14(1-63) were studied. All of them were found to be inactive, indicating that CXCL14-induced MRGPRX2 activation is associated with its C-terminal sequence (see Figs. 3b and c). In particular, the amino acids^47^ STKRFIK^48^ appeared to be important for CXCL14-MRGPRX2 interaction since CXCL14(1-56/64-77) was the largest inactive fragment, and it lacked that sequence (see Figs. 3b, c and e).

### Identification of the CXCL14 pharmacophore by testing small fragments

**Fig. 4:**
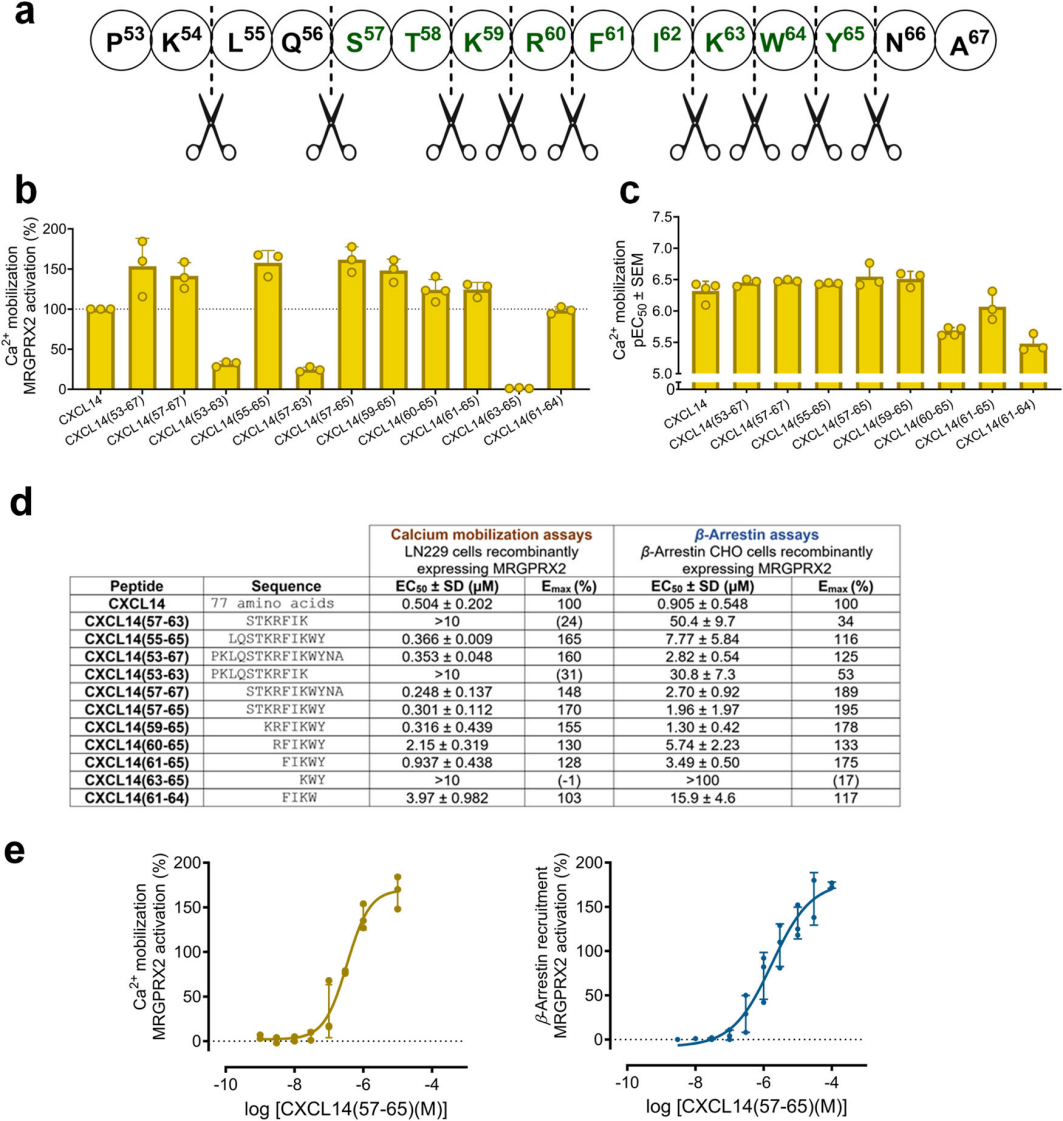
Identification of the pharmacophore of CXCL14 (small fragments). **a** Investigated partial peptide sequences of CXCL14. **b** pEC_50_ values of CXCL14 fragments determined in calcium mobilization assays. **c** Maximal effect of small CXCL14 fragments (10 µM) in calcium mobilization assays normalized to the effect of CXCL14 (1 µM). **d** EC_50_ and E_max_ values determined in calcium mobilization assays in comparison to values determined in *β*-arrestin recruitment assays. **e** Concentration-response curves of CXCL14(57-65) (PSB-231409A; STKRFIKWY) at MRGPRX2 in calcium mobilization assays (orange, EC_50_ 0.301 ± 0.112 µM), and *β*-arrestin recruitment assays (blue, EC_50_ 1.96 ± 1.97 µM). Data represent means ± SD from three to four independent experiments performed in duplicates. The effect was normalized to the maximal effect of CXCL14 (100%). Sigmoidal dose-response parameters (variable slope) were used to calculate EC_50_ and E_max_ values.

Based on the results obtained with larger partial CXCL14 sequences, a short peptide, CXCL14(57-63) consisting of only 7 amino acids, was designed and studied in both calcium mobilization and *β*-arrestin recruitment assays. The heptapeptide CXCL14(57-63) showed low potency and efficacy (EC_50_ > 10 µM in calcium assays; EC_50_ 50.4 µM, E_max_ 53% in *β*-arrestin recruitment assays) (see Fig. 4a). To improve the peptide-receptor interaction, two additional amino acids at both the C- and the N-terminus were added. In the resulting peptide CXCL14(55-65), MRGPRX2 activity was restored (see Figs. 4a, b). It is noteworthy that most of the small peptides were about 10-fold more potent in the calcium assay as compared to the *β*-arrestin assay. The calculated bias factors of the short fragments, ranging from 0.2 to 1.2, suggest a balanced activation of both systems with no significant ligand bias (see Supplementary Table for calculated bias factors of short peptides^49^). The shortest highly active peptide was CXCL14(61-65) consisting of only 5 amino acid residues, FIKWY (see Fig. 4b–d). A shorter peptide, the tetrapeptide FIKW (CXCL14(61-64)), still activated the receptor with similar efficacy as CXCL14, but with low potency (see Fig. 4d). The most potent small peptide with high efficacy was the nonapeptide CXCL14(57-65) (EC_50_ 0.301 µM, E_max_ 170%, calcium assays; EC_50_ 1.96 µM, E_max_ 195%, *β*-arrestin recruitment assays) (Fig. 4b–e).

### Computational studies

**Fig. 5:**
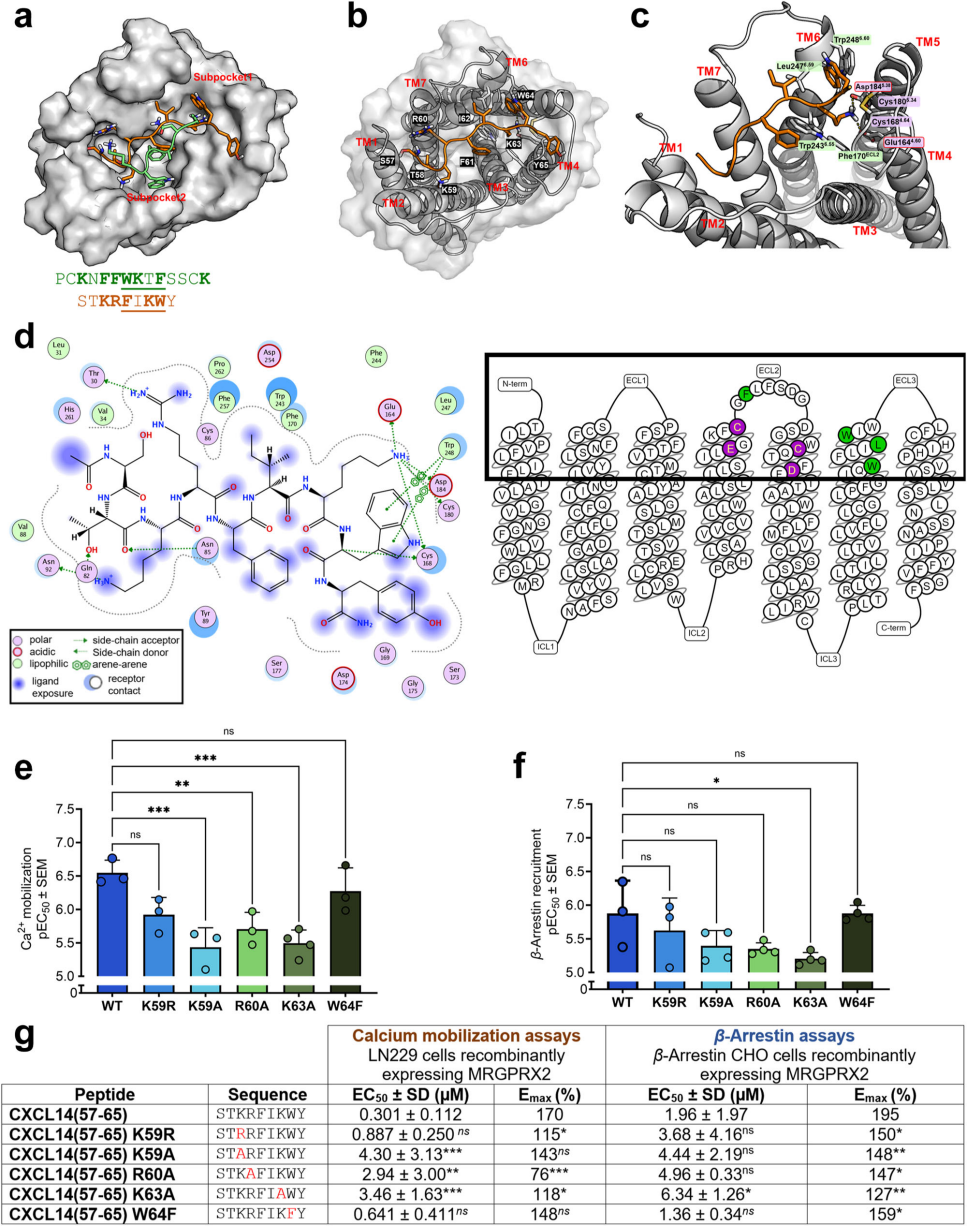
Investigation of the proposed peptide-receptor interaction site. **a** Cryo-EM structure of MRGPRX2 in complex Cortistatin-14 (PCKNFFWKTFSSCK with disulfide bridge: 2-13) in green color (PDB: 7S8L) superimposed by CXCL14(57-65) (STKRFIKWY) colored orange in its predicted binding pose, showing subpocket 1 and subpocket 2. The peptides that appear to be similar, FIKW and WKTF, are underlined in both sequences consisting of aromatic amino acid residues (F and W) framing two amino acid residues including a lysine (K). **b** Predicted binding pose of the peptide CXCL14(57-65). **c** Side-view of MRGPRX2 showing the transmembrane helices (TM1-TM7) and the suggested binding pocket of CXCL14(57-65), which is located near the surface of MRGPRX2 (shown in the snake plot of MRGPRX2). The amino acid residues glutamic acid (Glu164^4.60^), cysteine (Cys168^4.64^), phenylalanine (Phe170^ELC2^), cysteine (Cys180^5.34^), aspartic acid (Asp184^5.38^), tryptophan (Trp243^6.55^), leucine (Leu247^6.59^), and tryptophan (Trp248^6.60^) are shown; the amino acids in purple are polar, acidic amino acids are circled in red, and lipophilic amino acids are in green. **d** Two-dimensional view of CXCL14(57-65) docked into the predicted binding site of MRGPRX2 using the Molecular Operating Environment (MOE) software. A snake plot of MRGPRX2 is depicted and key amino acid residues important for interactions are highlighted. pEC_50_ values of wild type (WT) CXCL14(57-65) compared to mutant sequences K59R, K59A, R60A, K63A, and W64F, at MRGPRX2 measured (**e**) in calcium mobilization assays and **f** in *β*-arrestin recruitment assays. **g** EC_50_ and E_max_ values of the mutant peptides in both assay systems. All data are means ± SD of *n* = 3 to 5 biological replicates. The effects were normalized to the maximal effect of CXCL14 (1 µM, 100%). Sigmoidal dose-response parameters (variable slope) were used to analyze the data for calculating EC_50_ and E_max_ values. ^ns^ > 0.05; * ≤ 0.05; ** ≤ 0.01; *** ≤ 0.001 *p*-value (one-way ANOVA with Bonferroni’s multiple comparisons test). The amino acid residues of the receptor are labeled using the three-letter code and numbered according to the Ballesteros-Weinstein system^76^, while the amino acid residues of the tested fragments are designated using the one-letter code.

To investigate the interaction of CXCL14 with MRGPRX2, the potent small nonapeptide CXCL14(57-65) was used for docking studies. Recently, a cryogenic electron microscopy (cryo-EM) structure of MRGPRX2 in complex with the peptide agonist Cortistatin-14 was reported (PDB: 7S8L)^50^. This structure was utilized to perform docking studies with the newly identified peptidic agonist CXCL14(57-65). The putative binding pocket of the CXCL14-derived nonapeptide (PSB-231409A; STKRFIKWY) was comparable to that of Cortistatin-14 (PCKNFFWKTFSSCK, disulfide bridge: 2-13) (Fig. 5a). The predicted binding mode suggests that the peptide binds in the two described subpockets (Fig. 5a). Based on the analysis of the electrostatic potential surface, subpocket 1, formed by transmembrane helices 3-6 (TM3-6) and extracellular loop 2 (ECL2), is negatively charged, while subpocket 2, made up of TM1-3, TM6 and TM7, is predominantly lined by hydrophobic amino acids (Figs. 5b, c). CXCL14(57-65) is predicted to bind to the shallow binding pocket, and the lysine residue K63 of the nonapeptide likely forms strong electrostatic interactions with Glu164^4.60^ (TM4) and Asp184^5.38^ (TM5) of MRGPRX2. These amino acids are located close to Cys168^4.64^ (TM4) and Cys180^5.34^ (TM5), which may contribute to the formation of an inter-helix disulfide bond between TM4 and TM5 (Fig. 5e). An analogous binding mode was observed for the lysine residue in position 3 of the co-crystallized peptide Cortistatin-14^50^. The interaction of the CXCL14 pharmacophore CXC14(57-65) is probably further stabilized through hydrophobic interactions of its residues F61, I62, and W64 with Phe170^ECL2^, Trp243^6.55^, Leu247^6.59^, and Trp248^6.60^ of the receptor (Fig. 5c, d). Specifically, F61 may act as an anchor and gate-keeper residue between binding subpockets 1 and 2. The tetrapeptide FIKW is the smallest active fragment, which proves that these amino acid residues have major interactions with the target receptor (Fig. 4d). Framing this core sequence with basic and aromatic amino acids can stabilize the interactions leading to enhanced potency and efficacy as observed in our fragmentation studies (Fig. 4d).

To confirm the predicted interactions, mutagenesis of the agonistic sequence CXCL14(57-65) was performed. When K was mutated to A in the peptide sequence CXCL14(57-65)K63A, a significant decrease in potency was observed in both calcium and *β*-arrestin assay (Fig. 5e–g). The potency of the W64F mutant of CXCL14(57-65) maintained the same potency confirming the importance of a hydrophobic interaction. Removing W64 and Y65 from the peptide resulting in fragment CXCL14(57-63) led to a large decrease in potency (Fig. 4d). Preserving the hydrophobic residues F61, I62, and W64 as well as the electrostatic interaction of the peptide’s K63, gave the still potent small peptide CXCL14(61-65) FIKWY (Fig. 4d). However, cutting off F61 and I62 from the peptide sequence resulting in CXCL14(63-65), led to a complete loss in potency highlighting the importance of these residues (Fig. 4d). These findings support the predicted binding pose and interaction between the amino acid residues of the potent and highly efficacious CXCL14(57-65) and the binding pocket of MRGPRX2 (Fig. 5c).

It is, however, still unclear how the full-length CXCL14 interacts with the shallow MRGPRX2 binding pocket. An initial interaction might be followed by a conformational change to allow the binding of the pharmacophore. The exact binding mode can be addressed in a separate study, e.g. by obtaining a cryo-EM or X-ray structure of CXCL14 bound to MRGPRX2. In addition, mutagenesis studies are to be performed, exchanging the amino acid residues Glu164^4.60^, Asp184^5.38^ Phe170^ECL2^, Trp243^6.55^, Leu247^6.59^, and Trp248^6.60^, respectively, for alanine, in order to probe their role in interacting with CXCL14 and the CXCL14-derived small peptide agonists, such as CXC14(57-65).

### Activation of the putative MRGPRX2 mouse ortholog MRGPRB2 by CXCL14

**Fig. 6:**
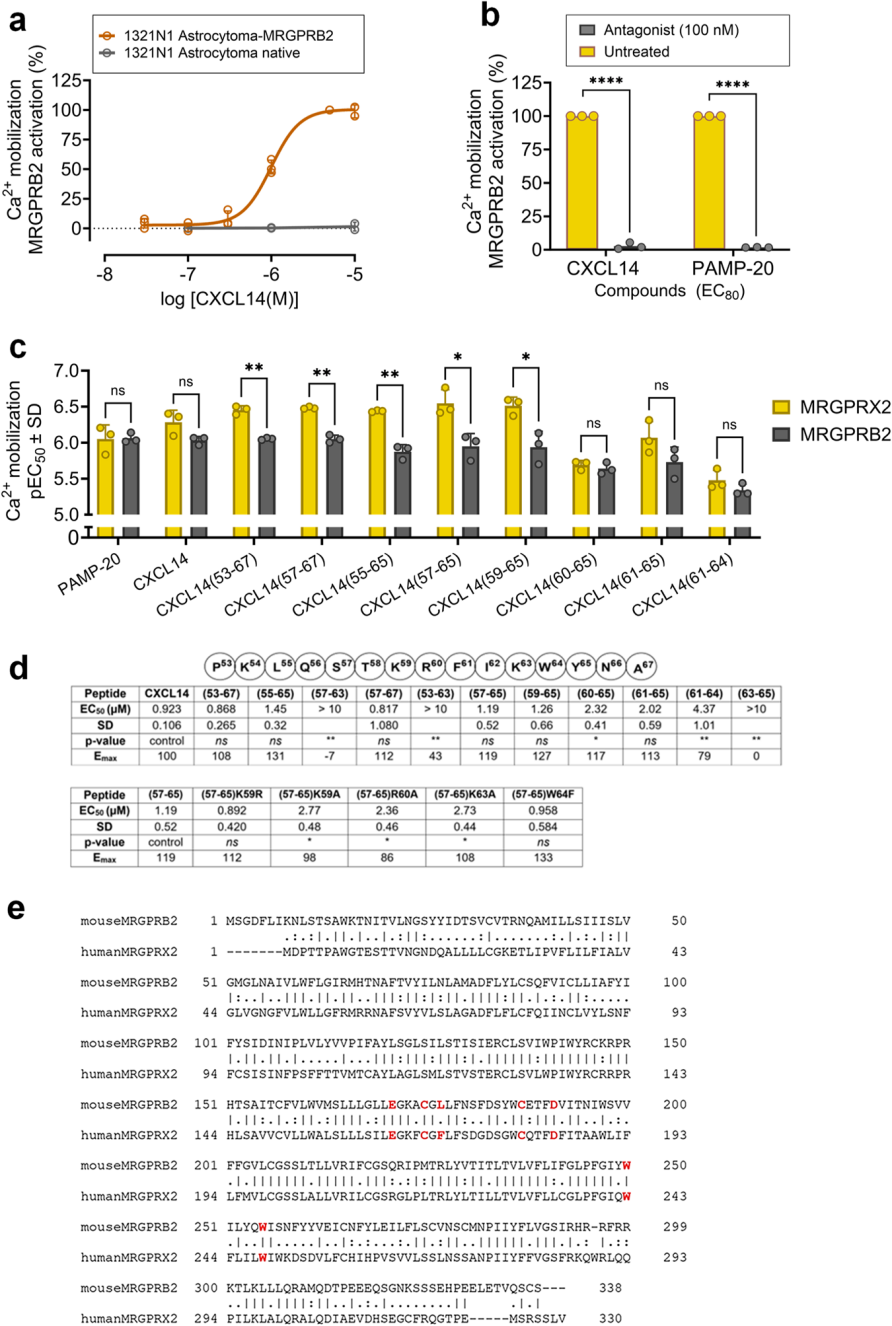
Activation of MRGPRB2 by CXCL14 and its fragments. **a** Concentration-response curves of CXCL14 at MRGPRB2 in calcium mobilization assays using 1321N1 astrocytoma cells stably expressing MRGPRB2 (CXCL14, EC_50_, 0.972 ± 0.105 µM) as compared to non-transfected 1321N1 astrocytoma cells. **b** CXCL14-induced MRGPRB2 activation (100 nM) is blocked by the antagonist PSB-172656 (1 µM) in calcium mobilization assays (**** ≤ 0.0005 *p* value). **c** Comparison of pEC_50_ values of CXCL14 fragments and mutants at MRGPRX2 and MRGPRB2 determined in calcium mobilization assays (multiple t-test). **d** EC_50_ and E_max_ values of CXCL14 fragments (compared to CXCL14) and mutants (compared to the peptide CXCL14(57-65)), not significant ^*ns*^ > 0.05; *≤0.05; **≤ 0.01 *p*-value (one-way ANOVA with Bonferroni’s multiple comparisons test). All data are means ± SD of *n*  =  3 to 4 biological replicates. **e** Overall sequence alignment between human MRGPRX2 and mouse MRGPRB2. Key amino acid residues forming interactions with the peptide CXCL14(57-65) are indicated in red. Vertical lines indicate conserved amino acids, one dot indicates non-conserved amino acids, and two dots indicate similar amino acids. Generated using the Clustal Omega1 sequence alignment tool.

CXCL14 is highly conserved among species, the mouse CXCL14 ortholog differing only by two of its 77 amino acids from the human chemokine^51^. In contrast, MRGPRX2 belongs to the primate-specific subfamily of MRGPRs^52^. However, the mouse MRGPRB2, belonging to a rodent-specific MRGPR subfamily, has been proposed to function as a mouse ortholog of the human MRGPRX2^53^. This was based on similar expression patterns, e.g. high expression on mast cells, and partly overlapping pharmacology^53^. Thus, we tested CXCL14 at the mouse MRGPRB2 stably expressed in 1321N1 astrocytoma cells using calcium mobilization assays. In fact, CXCL14 activated the mouse receptor (EC_50_ = 0.972 µM) (see Fig. 6a), while it failed to induce calcium mobilization in the non-transfected cell line (see Fig. 6b). Importantly, CXCL14-induced MRGPRB2 activation was blocked by the MRGPRX2/MRGPRB2 antagonist PSB-172656 (100 nM) (see Fig. 6b).

Next, the short CXCL14 fragments were tested at MRGPRB2, and concentration-response curves were established to determine EC_50_ values (see Fig. 6d). Like CXCL14, many of the small peptides showed potencies at MRGPRB2 similar to those observed at MRGPRX2 indicating that the binding pockets of both receptors are similar (see Fig. 6c). CXCL14(60-65), consisting of six amino acids, was found to be equipotent at both MRGPRB2 and MRGPRX2 (MRGPRB2, EC_50_ 2.32 µM, E_max_ 117%; MRGPRX2, EC_50_ 2.15 µM, E_max_ 130%) (see Fig. 6c), whereas the one amino acid longer peptide CXCL14(59-65) was more potent at MRGPRX2 (EC_50_ 0.316 µM, E_max_ 155%) than at MRGPRB2 (EC_50_ 1.26 µM, E_max_ 127%) (see Fig. 6d).

A sequence alignment of human MRGPRX2 and mouse MRGPRB2 is shown in Fig. 6e. The overall sequence similarity between the two receptors is ca. 62%, indicating significant conservation of amino acids between human MRGPRX2 and mouse MRGPRB2. Comparing the specific amino acids predicted to be involved in interactions with the peptide CXCL14(57-65) by docking studies, it is observed that Phe170 in MRGPRX2, which mediates hydrophobic interactions, is replaced by leucine (Leu161) in the mouse MRGPRB2. This may be the reason for the somewhat lower potency of CXCL14, and fragments consisting of more than 6 amino acid residues, at the MRGPRB2 as compared to the human receptor (Fig. 6c). Leucine is smaller and less hydrophobic than phenylalanine resulting in potential differences in protein conformation as well as hydrophobic interactions.

## Discussion

Herein we describe the discovery and validation of MRGPRX2 as a target of the proinflammatory orphan chemokine CXCL14. Among a large pool of 238 investigated GPCRs, MRGPRX2 was the only one activated by CXCL14 (1 µM) in *β*-arrestin recruitment assays, indicating the chemokine’s specificity for MRGPRX2, at least among the tested GPCRs (see Supplementary Data 2). Previous studies had claimed that CXCL14 may interact with GPR85, ACKR2, and CXCR4, although there have been some controversies^30–34^. In the present study we found that CXCL14 (1 µM) acts as an inverse agonist of CXCR4 blocking CXCL12-induced *β*-arrestin-2 recruitment (see Supplementary Data 2, Supplementary Fig. 2), whereas we observed no effect of CXCL14 at GPR85. Although ACKR2 was not included in our GPCR panel, it was previously reported that CXCL14 failed to induce *β*-arrestin-2 recruitment via ACKR2^29^. MAS-related G protein-coupled receptors (MRGPRs) belong to the δ-branch of the class A rhodopsin-like GPCR^54^. Mammalian MRGPRs are grouped into nine subfamilies, MRGPRA-H, and the primate-specific subfamily X, based on their sequence similarities. The MRGPR subfamily X consists of four orphan receptors, MRGPRX1, MRGPRX2, MRGPRX3, and MRGPRX4^52,55^. CXCL14 was found to be receptor subtype-selective since it did not activate the other MRGPRX subtypes, MRGPRX1, -3, and -4 even at a high concentration of 10 µM. MRGPRX2 can be activated by a number of basic molecules, positively charged at physiologic pH value of 7.4, including many drugs, thereby mediating pseudo-allergic reactions^52,56^. MRGPRX2 can also be activated by some peptides including cortistatin-14^57^, proadrenomedullin N-terminal peptide-12 (PAMP-12)^58^, PAMP-20^47^ and host defense peptides (antimicrobial peptides) such as cathelicidin LL-37 and *β*-defensins^59,60^. The receptor was reported to promiscuously couple to all G proteins with most robust coupling to G*α*_q_ and G*α*_i1_^61^. CXCL14-induced activation of the Gα_q/11_-coupled MRGPRX2 leads to canonical GPCR signaling as determined in calcium mobilization and G protein activation assays showing sub-micromolar potencies.

Circulating CXCL14 protein levels were previously determined using an immunoassay and found to be significantly elevated in plasma from IPF patients as compared to healthy controls (reaching almost nanomolar concentrations)^62^. Therefore, CXCL14 was suggested as a serum biomarker for monitoring the progression of fibrosis in IPF patients. Moreover, CXCL14 was demonstrated by histological staining of normal and IPF tissue sections to be highly expressed in IPF lung sections but not in normal lung^63^. Examining explanted lung tissue from IPF patients revealed that CXCL14 protein and gene expression are specifically localized to fibrotic regions while being virtually absent in healthy areas^62^. Whereas it remains uncertain if excess plasma CXCL14 in IPF patients stems from lung tissue, data from tumor patients before and after treatment indicated that increased plasma CXCL14 levels originated from the lung^62^. These previous reports suggest that, although the measured CXCL14 serum concentrations would not be sufficient to activate MRGPRX2 (EC_50_ 504 nM), local concentrations of the chemokine can be expected to reach the threshold to activate MRGPRX2. However, future research using an MRGPRX2 antagonist in an appropriate IPF mouse model will be necessary to elucidate the role of CXCL14/MRGPRX2 in this disease. Initially, MRGPRX2 was found to be expressed in dorsal root ganglia (DRG)^54^. The subsequent discovery of MRGPRX2 expression and the receptor’s role in mast cells, basophils and eosinophils has been considered one of the most significant breakthroughs in the field of allergy and immunology in the past years^35,36^. Chymase/tryptase-containing skin mast cells (MC_TC_) express higher levels of MRGPRX2 than tryptase-containing lung mast cells (MC_T_)^37^. However, in chronic inflammatory lung diseases, MRGPRX2 expression is significantly increased in lung mast cells compared to healthy controls^37^. MRGPRX2 activation leads to mast cell degranulation and thereby contributes to allergic and inflammatory diseases. CXCL14 and MRGPRX2 expression overlap in many diseases e.g. asthma^20,37^, rheumatoid arthritis^23,38^, and atopic dermatitis^24,39^. Additionally, they share similar expression patterns in skin and skin mast cells^10,35^.

Our efforts to identify the pharmacophoric structure of CXCL14 revealed that only 4 out of 77 amino acids, namely FIKW in the C-terminal *α*-helix of CXCL14, are required for full MRGPRX2 activation. Lu et al. proposed that MRGPRX2-activating peptides share a common motif consisting of an aromatic amino acid, at least 3 amino acids containing one or more positively charged amino acids, and a hydrophobic amino acid^48^. Our work identified the tetrapeptide FIKW as the minimal pharmacophoric structure of CXCL14, which is a new finding since it is even smaller than the previously suggested pentapeptidic motif.

CXCL14 possesses this motif in its C-terminal part thereby exposing the pharmacophore for interaction with MRGPRX2. Other CXCLs contain sequences that are similar to the proposed pentapeptidic motif, but are either deeply buried in the typical 3D structure of the chemokine, or located near acidic amino acids, that are not tolerated by the negatively charged binding pocket of MRGPRX2 (see sequence alignment in Supplementary Fig. 4 for further information). C-terminal aromatic residues adjacent to basic residues, but not to acidic residues, are only found in CXCL4, CXCL13, CXCL14, and CXCL17. Notably, CXCL4, also known as platelet factor 4, was previously reported to weakly activate MRGPRX2 recombinantly expressed in HEK293 cells (EC_50_ value of 9 µM in calcium mobilization assays)^35^. Upon checking the sequence of CXCL4, one might speculate that the compound’s activity resides in the C-terminal sequence YKKIIK, but this still needs to be confirmed.

Chemokines typically bind to their receptors via a two-step/two-site mode^64^. Chemokine recognition site 1 is located on the N-terminus of the chemokine receptor that interacts with the globular core of the chemokine^64^, while chemokine recognition site 2 is located within the receptor transmembrane domains and interacts with the N-terminus of the chemokine^64^. Our findings show that the interaction of CXCL14 with MRGPRX2 is very different; e.g., only a small partial structure of CXCL14 is sufficient, and its N-terminus is not required for MRGPRX2 binding and activation. Other chemokines have also been reported to deviate from the traditional two-site binding paradigm^64^.

Some of the small CXCL14 fragments comprising the tetrapeptidic pharmacophore ^61^FIKW^64^ (designated PSB-231404) showed super-agonistic activity in calcium mobilization and *β*-arrestin recruitment assays compared to CXCL14. These short peptides lack the cysteine residues required for disulfide bond formation and 3D-folding. Therefore, fragments containing the exposed pharmacophore appear to have maximal interaction with MRGPRX2 resulting in increased efficacy compared to the full chemokine sequence. The highest efficacy was achieved by the nonapeptide STKRFIKWY (CXCL14(57-65), PSB-231409, E_max_ 170% in calcium assays, 195% in *β*-arrestin assays, compared to CXCL14 set at 100%). This peptide was used in docking studies to predict its interactions within the MRGPRX2 binding pocket. CXCL14(57-65) contains three positively charged amino acids in positions 59, 60, and 63. Exchanging any of these basic amino acids for alanine resulted in reduced potency and efficacy. Basic amino acids of CXCL14 are predicted to interact with the negatively charged amino acid residues Glu164 and Asp184 in the MRGPRX2 binding pocket, and the interactions are likely further stabilized by binding of the ligand’s amino acid residues F61, I62, and W64 with the receptor’s Phe170, Trp243, Leu247, and Trp248 through hydrophobic interactions. Our experimental studies revealed that the full-length CXCL14 showed similar potency as some of its fragments. Thus, the typical two-step binding of other chemokines at chemokine receptors^64^ appears to be very different from the binding mode of CXCL14 at MRGPRX2. Conformational changes of both MRGPRX2 and CXCL14 may be involved, facilitating the interaction of the C-terminal binding motif of CXCL14 with the shallow binding pocket of MRGPRX2 (see PDB: 7S8L)^50^. Only additional studies, e.g. a cryo-EM or an X-ray structure of the CXCL14-MRGPRX2 complex, or mutagenesis studies on the receptor, would eventually provide concrete insights into the binding mode of the protein-ligand. Because several of the short CXCL14 fragments are potent super-agonists, it may be speculated that the expression of CXCL14 along with an appropriate set of proteases (see protease activities in Supplementary Fig. 5) may lead to CXCL14 cleavage turning the agonist into a potent super-agonist. This might play a role in pathologic conditions, such as IPF^17–19^, obstructive pulmonary disease (COPD), lung cancer^16^, and asthma^20^. Human proteases like neutrophil elastase are predicted to digest CXCL14 at five positions, namely 17, 34, 41, 67, and 74, producing, among others, a CXCL14 fragment with the sequence STKRFIKWY that can activate MRGPRX2 with high efficacy. Similarly, *staphylococcal* peptidase I digest CXCL14 in positions 21, 30, 48, 70, and 76, presumably resulting in the release of potent and efficacious MRGPRX2-activating peptide fragments. It should be noted that CXCL14 was reported to have antimicrobial activity against gram-negative and gram-positive skin-associated and respiratory tract bacteria^22^. Further studies reported that the C-terminal *α*-helix of CXCL14 and its analogs have a dual effect, an antimicrobial effect that is mediated via bacterial membrane disruption and bacterial DNA binding, and an anti-inflammatory effect observed in a mouse macrophage cell line^65^. According to the present findings, this latter effect may be explained by activation of the proposed MRGPRX2 mouse ortholog, MRGPRB2. The mouse MRGPRB2 shares a similar expression pattern and some ligands with MRGPRX2, although both receptors display only moderate sequence similarity (65%)^53^.

The short tetrapeptide FIKW (PSB-231404, CXCL14(61-65)) and some of its derivatives, in particular, the nonapeptide STKRFIKWY (PSB-231409, CXCL14(57-65)) and the pentapeptide FIKWY (PSB-231405, CXCL14(61-65) could be used as easily accessible, inexpensive tool compounds to study whether the various reported effects of CXCL14 are due to MRGPRX2/MRGPRB2 receptor activation. Moreover, these compounds could be employed for target validation studies in animal models of IPF and further inflammatory diseases.

Previously, an effect of CXCL14 on mast cells was described, namely partial degranulation leading to the secretion of de novo synthesized CXCL8 via an unknown mechanism^66^. Here we report a direct activation of MRGPRX2, which is expressed in mast cell, basophils, and eosinophils, by CXCL14 and some of its fragments^35,36^, providing a possible molecular mechanism for CXCL14-induced mast cell activation. Given the established methodologies, further exploration into the reported effect of CXCL14 on mast cells via MRGPRX2 antagonism and the study of its fragments remains to be addressed in the future. Earlier studies reported that CXCL14-deficient mice do not exhibit clear immune system abnormalities suggesting its potential role only in disease or upon stimulation^67^. We showed that MRGPRB2, the proposed functional ortholog of MRGPRX2 in mice^53^, is activated by CXCL14 at similar concentrations as the human MRGPRX2 (MRGPRX2, EC_50_ 0.504 µM; MRGPRB2 0.923 µM). This chemokine is highly conserved among both species, differing in only two amino acids in its N-terminus. The identified pharmacophoric sequence of CXCL14 responsible for MRGPRX2/B2 activation is conserved between human and mouse. Despite the low similarity between both receptors, MRGPRX2 and MRGPRB2, the proposed binding pocket for CXCL14 appears to be conserved. MRGPRX2 as well as MRGPRB2 activation by CXCL14 was inhibited by the selective MRGPRX2/B2 antagonist PSB-172656. This will facilitate future investigations of CXCL14-induced effects on MRGPRs since mouse models can be employed.

In conclusion, we discovered MRGPRX2 as a target of the orphan proinflammatory chemokine CXCL14 both of which are upregulated in pulmonary fibrosis. We identified the essential activating motif of CXCL14, which displays super-agonistic properties at MRGPRX2 and also activates its functional mouse ortholog MRGPRB2.

The identification of the CXCL14 receptor that is expressed on mast cells and other MRGPRX2-expressing cells and tissues will facilitate studying and investigating its role in inflammatory diseases such as fibrosis, asthma, arthritis, and skin diseases. Furthermore, it offers a promising prospect for the development of new therapeutic approaches targeting IPF and further inflammatory diseases. In addition, our results provide a basis for the development of small peptides or peptidomimetics with MRGPRX2-agonistic activity as a targeted therapeutic intervention for the treatment of bacterial infections.

## Methods

### Reagents and peptides

CXC motif chemokine 14 (CXCL14) was purchased from BIOZOL (Eching, Germany). Proadrenomedullin N-terminal 20 peptide (PAMP-20) was obtained from Tocris Bioscience (Bristol, UK), and cortistatin-14 was from GenScript (NJ, USA). Custom synthesis of CXCL14 analogs was performed by JPT Peptide Technologies (Berlin, Germany) (see Supplementary Fig. 6). Stock solutions were prepared either in dimethyl sulfoxide (DMSO) or in RNase-, DNase- and protease-free water from Carl Roth GmbH & Co (Karlsruhe, Germany). The MRGPRX2 antagonist PSB-172656 (synonymous designations: B-40 and CB70) was synthesized as descibed^68,69^.

### Plasmids

The constructs pLXSN-MRGPRX2 (NM_001303615.2), pLVX MRGPRB2-IRES-mCherry (NM_175531.4), and pCMV-ARMS1-ProLink2^TM^-MRGPRX3 (NM_054031.3) had been generated as previously described^69,70^. All plasmids were verified by sequencing (Eurofins Genomics Germany, Ebersberg, Germany).

### Cell lines and transfection

**The cell lines CHEM1 and CHEM1-MRGPRX2** are adherent rat cell lines (HTSCHEM-1RTA, HTS058RTA, Merck-Millipore, Darmstadt, Germany); which were cultured under standard cell culture conditions.

**LN229 glioblastoma cells ATCC**^**®**^, CRL-2611TM, were a kind gift from Prof. Dr. B. Scheffler (University of Bonn, Germany). LN229 cells stably expressing MRGPRX2 were generated by retroviral transfection. Briefly, GP^+env^AM-12 packaging cells (LGC Standards GmbH, Wesel, Germany), derived from mouse fibroblasts, were seeded at a density of 1.5 × 10^6^ cells per T25-flask in 5 mL of Dulbecco’s Modified Eagle Medium (DMEM, Thermo Fisher Scientific, Waltham, MA, USA), supplemented with 10% fetal calf serum (FCS), 100 U/mL of penicillin (PAN-Biotech) and 100 µg/mL of streptomycin (PAN-Biotech). Packaging cells were transfected with pLXSN-MRGPRX2 and pcDNA3-VSV-G using Lipofectamine 2000 according to the manufacturer’s instructions (Thermo Fisher Scientific, Darmstadt, Germany). The medium was exchanged for DMEM supplemented with 10% FCS, 100 U/mL of penicillin, 100 µg/mL of streptomycin and 3 mM of sodium butyrate (stock solution: 500 mM in water, sterile-filtered) to enhance virus production. To produce retroviral particles, cells were cultivated for 48 h at 32°C and 5% CO_2_. On day 4, the target cells LN229 were seeded into T25 cell culture flasks (5 × 10^5^ cell). The supernatants of the packaging cells (3 mL) containing infectious retroviral particles were sterile-filtered on the next day using Filtropur S 0.2 µm non-pyrogenic sterile filters (Sarstedt AG & Co. KG, Nümbrecht). Polybrene (hexadimethrine bromide, a cationic polymer, stock solution 4 mg/mL in water, sterile-filtered), was added to the supernatant (final concentration: 0.008 mg/mL). The medium of the LN229 cells was removed and exchanged for the supernatant containing the polybrene solution. After 150 min, the medium was changed back to DMEM medium containing 10% FCS, 100 U/mL of penicillin and 100 µg/mL of streptomycin. Cells were selected after 48 h by the addition of G418 (200 µg/mL).

**1321N1 Astrocytoma cells** were purchased from Sigma Aldrich-Merck KGaA (Darmstadt, Germany, 86030402). The cells were transfected with MRGPRB2 using lentiviral transfection. 1.5 × 10^6^ Lenti-X 293 T cells were seeded into each T25 cell culture flask in 5 mL of DMEM, supplemented with 10% FCS, 100 U/mL of penicillin and 100 µg/mL of streptomycin. After 20 h of incubation at 37°C and 5% CO_2_, when the cells were grown to 80–90% confluence, the medium was exchanged for 5 mL DMEM with 10% FCS without antibiotics and incubated for 4 h. Subsequently, transfection took place using Xfect polymer according to the manufacturer’s protocol (Clontech, Takara Bio Europe, France). The pLVX MRGPRB2-IRES-mCherry-Xfect mixture was added to the packaging cells and they were incubated overnight. Two days post-transfection, the supernatant from transfected Lenti-X cells containing the lentiviral particles were harvested, filtered, and then added to the target cells, 1321N1 astrocytoma cells, with 6 µL polybrene solution (4 mg/mL in water, final concentration: 0.008 mg/mL). After 150 min of lentiviral transfection the virus containing medium was removed and replaced for 5 mL of DMEM, supplemented with 10% FCS and 100 U/mL of penicillin and 100 µg/mL of streptomycin. Cells were incubated for 72 h at 37°C and 10% CO_2_. When the cells showed 80% confluence, they were seeded into Nunc™ 177399 Lab-Tek® Chamber Slide™ Systems. To know how many astrocytoma cells expressed the target receptor, cells were analyzed under the fluorescence microscope at the m-cherry excitation wavelength of 587 nm using its emission wavelength of 610 nm. Subsequently, fluorescent single clones were isolated by fluorescence-activated cell sorting.

**Chinese hamster ovary (CHO)** ***β*-arrestin cells** had been engineered by Eurofins DiscoverX (Fremont, CA, USA) to stably express *β*-arrestin-2 fused to a large fragment of *β*-galactosidase (called enzyme acceptor, or EA). These commercially available parental CHO-K1-*β*-arrestin-2 cells (93-0164) were purchased. Additionally, CHO *β*-arrestin cells expressing MRGPRX1, MRGPRX2 and MRGPRX4 fused to a small enzyme donor fragment ProLink™ (PK) were purchased (93-0919C2, 93-0309C2 and 3-0541C2A, respectively). Parental CHO-*β*-arrestin cells were cultured in a humidified incubator with 5% CO_2_ at 37°C in Gibco F-12 Ham Nutrient Mixture medium (Life Technologies) supplemented with 10% FCS, 100 U/mL of penicillin, 100 µg/mL of streptomycin, and 300 μg/mL of hygromycin. CHO-*β*-arrestin cells expressing MRGPRX3 were established by transfecting the parental CHO-K1-*β*-arrestin-2 cells with a pCMV-ARMS1-ProLink2^TM^-MRGPRX3 plasmid using Lipofectamine^TM^ 2000 (Thermo Fischer Scientific, Schwerte, Germany) according to the manufacturer’s recommendation, and stably expressing cells were obtained by antibiotic selection with gentamicin (PAN-Biotech). Cells expressing the desired receptor were then maintained in 800 μg/mL gentamicin.

### Calcium mobilization assays using CHEM-1 cells

A cell-based fluorometric assay was employed for the determination of intracellular calcium release using Fluo-8. Cells were seeded into microtiter plates and incubated under standard cell culture conditions. After 24 h the medium was removed, and the cells were washed with phosphate-buffered saline (PBS). The cells were incubated with loading buffer (40 ml PBS with 2 mM Ca^2+^, 800 µl of brilliant black solution (2%), 732 µl of probenecid solution (0.5 M) in 1 N NaOH, 400 µL of pluronic (10%) in water, and 152 µl of Fluo-8 (5.7 ng/ml in water)) for 30 min. For fluorescence readout and compound testing a FLIPR tetra system was used according to the manufacture’s protocol (Molecular Devices, CA 94089- 1136, USA).

### Calcium mobilization assays using LN229 cells

On the day before the assay, glioblastoma LN229-MRGPRX2 stably expressing MRGPRX2 (produced by retroviral transfection) were seeded into black sterile 96-well microplates at a density of 2.5 × 10^5^ cells/mL per well in 200 µL of DMEM/F12- Nutrient Mixture medium, supplemented with 10% FCS, 100 U/mL of penicillin 100 µg/mL of streptomycin, and 200 µg/mL of gentamicin, and incubated overnight. Fluo-4 AM solution (1 mM) (Thermo Fisher Scientific) was prepared by diluting 50 µg in 45.9 µL of DMSO. On the day of the assay, the cell medium was removed and exchanged for 40 µL of dye solution per well, consisting of 4970 µL of Hank’s Buffered Saline Solution (HBSS), 15 µL of 20% Pluronic-F and 15 µL of 1 mM Fluo-4 AM (final concentration of Fluo-4 AM: 3 µM). After an incubation time of 1 h at room temperature in the dark, the solution of each well was replaced by 190 µL of HBSS. Agonist solution (10 µL) in HBSS, namely carbachol (100 µM), or PAMP-20 (5 µM), was pipetted using a FlexStation® 3 Multi-Mode Microplate Reader. For the determination of baseline fluorescence, HBSS buffer without additives was used. Fluorescence was measured at 525 nM (excitation 485 nM) for 120 intervals of 1 s (number of flashes: 10 each) by utilizing the SoftMax® Pro5.1 Microplate Data Acquisition & Analysis Software. Three to four independent experiments were performed in duplicates unless otherwise indicated. Measurements are given in relative fluorescent units as the maximum response minus the minimum response.

For testing the MRGPRX2/B2 antagonist PSB-172656, similar steps were carried out except that the dye solution was replaced by 189 µL of HBSS, and the MRGPRX2/B2 antagonist was diluted in DMSO, then added manually (1 µL, final concentration 100 nM) 30 min before agonist addition as described above. The final DMSO concentration did not exceed 0.5% (v/v).

### Calcium mobilization assays using 1321N1 astrocytoma cells

1321N1 astrocytoma cells recombinantly expressing MRGPRB2-mCherry were used. All other steps were as described above for MRGPRX2, except that the fluorescence was measured at 525 nM (excitation 485 nM) for 60 intervals of 1 s (number of flashes: 10 each) by utilizing the SoftMax® Pro5.1 Microplate Data Acquisition & Analysis Software.

### *β*-Arrestin recruitment assays

*β*-Arrestin recruitment assays were based on the enzyme complementation technology. The assays were carried out as previously described^56^. Briefly, cells were seeded into white 96-well plates (Nunclon Delta surface plates, Thermo Fisher Scientific) in 90 µl Opti-MEM medium (Thermo Fisher Scientific) supplemented with 2% fetal bovine serum, 100 U/mL of penicillin, 100 µg/mL of streptomycin, 800 μg/mL of gentamicin, and 300 μg/mL of hygromycin B. On the next day, the assays were performed. Peptides were diluted, and 10 µl of solution were added to the cells, which were subsequently incubated for 90 min. The final DMSO concentration did not exceed 1% (v/v). Then, 50 µL of detection reagent (DiscoverX) was added. After 60 min of incubation in the dark at room temperature, chemiluminescence was measured by using a Mithras LB 940 plate reader (Berthold Technologies, Bad Wildbad, Germany). The results are means ± SDs of three to five independent experiments, each in duplicates.

### GPCR panel screening

Screening the GPCRs panels was performed by DiscoverX (Eurofins) using *β*-arrestin recruitment assays unless otherwise indicated (for details, see Supplementary Data 2).

### Immunostaining of MRGPRX3-expressing cells

One day before staining, *β*-arrestin-CHO-MRGPRX3 cells were seeded onto sterile coverslips in a 6 well-plate (Sarstedt, Nümbrecht, Germany) and cultured overnight. The next day, the cells were fixed with 4% paraformaldehyde (pre-heated at 37 °C) for 20 min at room temperature. The cells were then washed with phosphate-buffered saline (PBS, pH 7.4) and blocked for 15 min with 1% bovine serum albumin/PBS solution. Cells were incubated in the dark for 60 min with the primary antibody, and then 30 min with the secondary antibody both diluted in 1% BSA/PBS. The primary antibody was diluted at 1:1000, and the secondary antibody at 1:500). PBS was used to wash cells between and after antibody incubations. In addition, the nuclei of the cells were stained with 4′,6-diamidino-2-phenylindole (DAPI) (1 mg/μl diluted 1:1000 in 1% BSA/PBS) for 5 min in the dark. Finally, coverslips were mounted using Fluoromount^TM^ Aqueous Mounting medium (Sigma-Aldrich-Merck KGaA, Darmstadt, Germany) and stored in the dark at 4°C. The mouse monoclonal anti-PK/PL antibody (DiscoverX, Fremont, CA, USA) was used as the primary antibody and goat anti-mouse-AlexaFluor488 (Jackson Immuno Research, Hamburg, Germany) as the secondary antibody to stain the Prolink tag on MRGPRX3. A Nikon A1 spectral confocal microscope operating with an argon laser, and the NIS Element Advanced Research software 4.0 were used for image acquisition and analysis. Each staining was repeated two to three times and at least ten squares (60× objective), each containing 3–15 cells, were imaged for each sample. Representative pictures are shown (Supplementary Fig. 1b).

### Molecular modeling

The cryoEM structure of the human MRGPRX2 in complex with the peptide ligand cortistatin-14 was selected for molecular docking experiments using AutoDock PDB: 7S8L^50,71^. A model of the human MRGPRX2 was prepared using the protein preparation tool, and protonated according to Protonate 3D implemented in Molecular Operating Environment (MOE 2019.01)^72^. The AutoDockTools were applied to add the atomic partial charges and for computing the three-dimensional energy scoring grid for a box of 60 × 60 x 60 points with a spacing of 0.375 Å^71^. The prepared structure of the human MRGPRX2 was applied for flexible molecular docking using AutoDock4.2. The selected peptide CXCL14(57-65) was docked into the binding site of the peptide agonist cortistatin-14, previously identified by cryoEM of MRGPRX2^47,50^ Fifty independent docking calculations were performed using the *var*CPSO-ls algorithm from PSO@Autodock implemented in AutoDock4.2^71^. The docking calculations were computed by setting the termination criteria as 50,000 evaluation steps. The parameters “cognitive coefficient (c1)” and “social coefficient (c2)” of the *var*CPSO-ls algorithm was set at 6.05 and the swarm size at 60 individual particles. The default values were applied for the remaining parameters of the algorithm. The poses obtained for CXCL14(57-65) from docking studies were explored by visual inspection on the basis of the docking score and the top-ranked pose was selected as the putative binding pose. For sequence alignment, the Clustal Omega online tool was used^73^. The snake plot of MRGPRX2 was generated using the GPCR database^74^.

### G protein activation assay using BRET^2^ (TRUPATH)

MRGPRX2-induced G protein heterotrimer dissociation was measured in LN229 cells using a BRET^2^ assay (TRUPATH, Addgene kit no. 1000000163)^45^, in analogy to a previously described procedure with minor modifications^75^. On the first day, parental LN229 cells, or LN229 cells stably overexpressing MRGPRX2, cultivated in a growth medium, were detached from cell culture flasks by trypsination. Cells were seeded into 6-well plates at a density of approximately 700,000 cells per well in a volume of 2 mL, and incubated at 37 °C for 4 h before transfection. Transient transfection was performed using Lipofectamine 2000 according to the manufacturer’s recommendation (ThermoFisher, Waltham, MA, USA). The cells were then transfected with the biosensors (100 ng of each pcDNA5/FRT/TO-G*α*_i1_-RLuc8, pcDNA3.1-G*β*_3_, and pcDNA3.1-G*γ*_9_-GFP2 per well) and incubated at 37 °C for 18 h. On the second day, media were removed, and cells were gently detached by adding 0.1 M phosphate-buffered saline, pH 7.4, containing 0.5 mM ethylenediaminetetraacetic acid (EDTA), and transferred to 96-well white bottom plates (Greiner BioOne, Frickenhausen, Germany) at a density of 30,000 cells per well in 60 μL of growth medium. On the third day, the assay was performed by careful aspiration of the medium and by gently washing the cells with assays buffer (HBSS and 20 mM 4-(2-hydroxyethyl)-1-piperazineethanesulfonic acid (HEPES), pH 7.4). Subsequently, assay buffer was added (60 µL) followed by 10 µL of a freshly prepared 50 µM coelenterazine 400a solution (Biomol, Hamburg, Germany). After a 5 min equilibration period, 30 μL of agonist solution was added to the cells and the mixture was incubated for 5 min. The final DMSO concentration in the assay buffer was 1%. Next, luminescence and fluorescence were measured in a Mithras LB940 plate reader, using 395 and 510 nm emission filters for the RLuc8 and GFP2 signals, respectively. The BRET ratio between the GFP2 and the RLuc8 signal intensity was computed and corrected for the baseline signal to obtain ΔBRET values. Results were visualized by GraphPad PRISM 8 (GraphPad, San Diego, CA, USA).

### Statistics and reproducibility

Data were analyzed using Prism 10.0 (GraphPad Software Inc., San Diego, CA, USA). All data are presented as means ± SD from three to five independent experiments performed in duplicates unless otherwise indicated. The graphs were generated using the same software. Sigmoidal dose-response (variable slope) parameters were used for analyzing the data to calculate EC_50_ and E_max_ values. To determine the level of significance, data were analyzed using one-way ANOVA with Bonferroni’s multiple comparisons test and multiple t-tests with Welch correction were used for pairwise comparisons between two groups (Prism 10.0). A *p*-value of less than or equal to 0.05 was considered to be significant and exact *p*-values are disclosed in a Source Data file (Supplementary Data 3). The NIS Element Advanced Research software 4.0 was used for microscopy image acquisition and analysis.

### Reporting summary

Further information on research design is available in the Nature Portfolio Reporting Summary linked to this article.

### Supplementary information

Supplementary Information

Description of Additional Supplementary Files

Supplementary Data 1

Supplementary Data 2

Supplementary Data 3

Reporting Summary

## Data Availability

The data supporting the findings of this study are available in Supplementary Data 3. Further information is available upon reasonable request from the corresponding author. IPF gene expression data can be obtained from the Gene Expression Omnibus database (GEO accession number: GSE2052) (https://www.ncbi.nlm.nih.gov/geo/). The PDB files of the docked peptides are provided in the supplementary information. All new plasmids were deposited with Addgene: pLXSN-MRGPRX2 (Plasmid #213960), pLVX-IRES-MRGPRB2-mCherry (Plasmid #213961), and pCMV-ARMS1-ProLink2-MRGPRX3 (Plasmid #213962).
