# Supplementary material for: Proinflammatory chemokine CXCL14 activates MAS-related G protein-coupled receptor MRGPRX2 and its putative mouse ortholog MRGPRB2
Source: Commun Biol. 2024 Jan 6;7:52. doi: 10.1038/s42003-023-05739-5 (PMC10771525; doi:10.1038/s42003-023-05739-5)
Supplement: Supplementary file 1 — Supplementary Information [file 42003_2023_5739_MOESM1_ESM.pdf]

Proinflammatory chemokine CXCL14 activates MAS-related G protein-coupled receptor MRGPRX2 and its putative mouse ortholog MRGPRB2

Ghazl Al Hamwi[1], Vigneshwaran Namasivayam[1], Beatriz Büschbell[1], Robin Gedschold[1], Stefan Golz[2], Christa E. Müller[1]*

[1]PharmaCenter Bonn, Pharmaceutical Institute, Pharmaceutical & Medicinal Chemistry, University of Bonn, An der Immenburg 4, 53121 Bonn, Germany

[2]Lead Identification & Characterization, Pharma Research and Development Center, Bayer AG, Wuppertal, Germany

Corresponding author: Christa E. Müller (christa.mueller@uni-bonn.de)

Supplementary Information

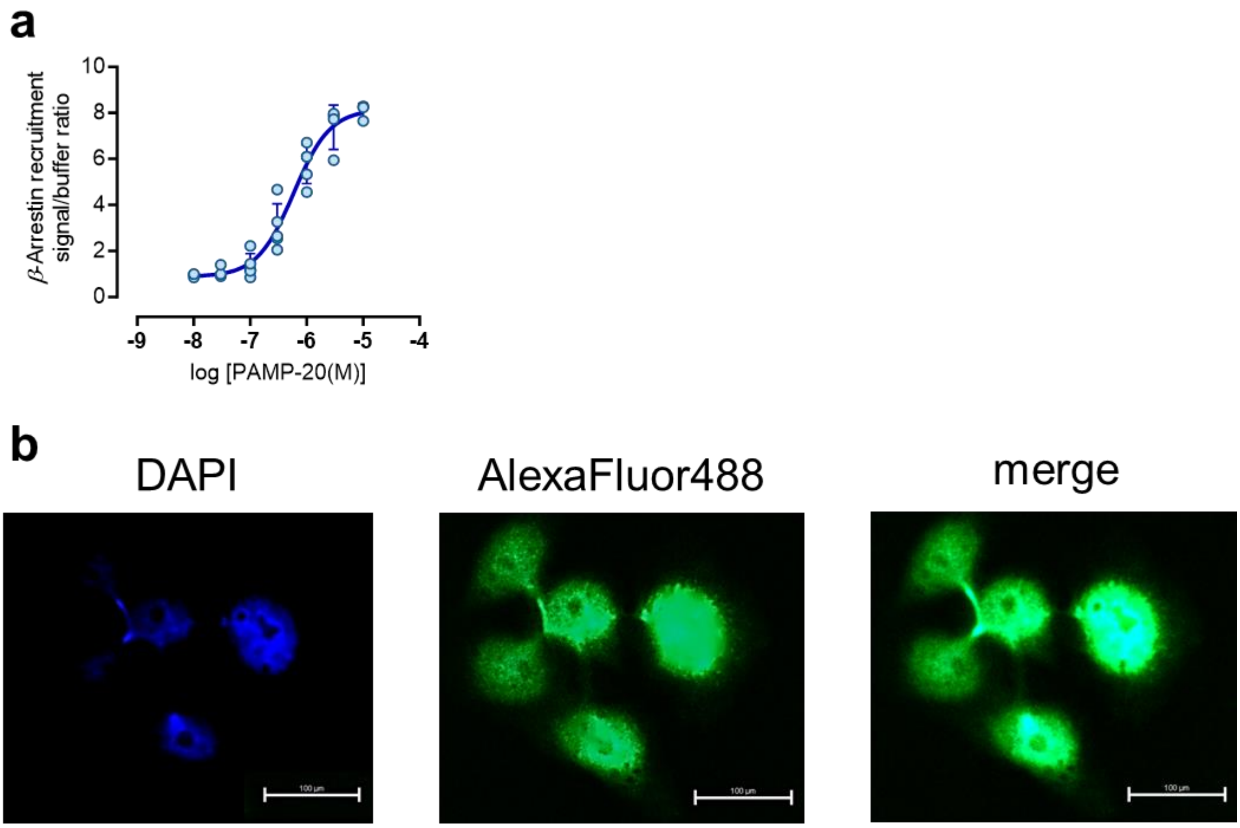

Supplementary Fig. 1: Control experiments for MRGPRX subtypes. a Concentration-response curve of PAMP-20 in *β*-arrestin-CHO cells recombinantly expressing MRGPRX2 measuring *β*-arrestin recruitment (PAMP-20, EC_{50} 1.08 ± 0.53 µM). **b** Imunostaining of *β*-arrestin-CHO cells recombinantly expressing MRGPRX3. Nucleus: DAPI; receptor: ProLink-tag (DiscoverX), fused to the receptor (1st antibody) and Alexa488 (2nd antibody). The scale bar is 100 µm. Controls for MRGPRX1 and MRGPRX4 are collected in Supplementary Data 2.

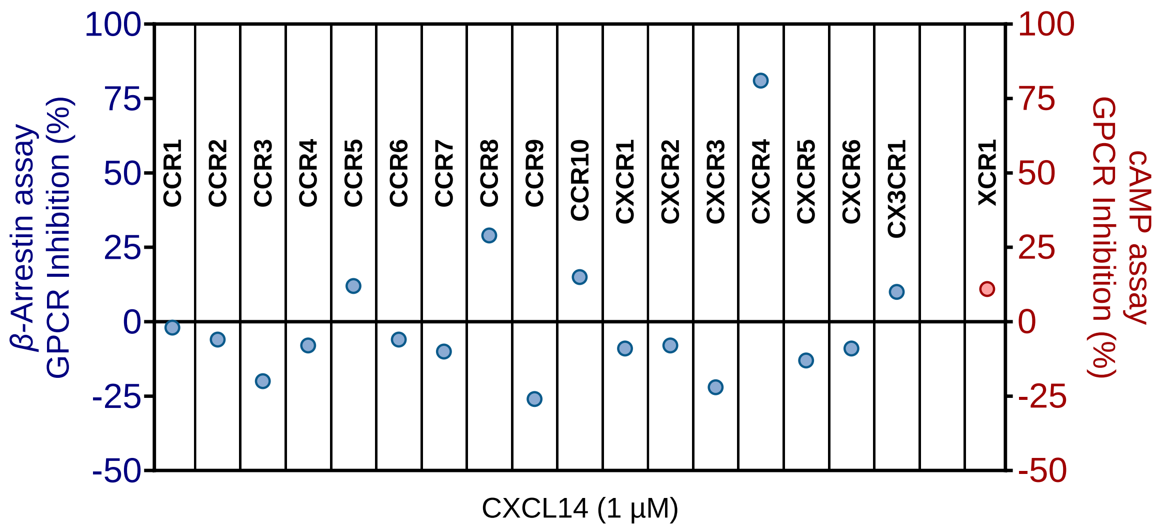

Supplementary Fig. 2: Screening CXCL14 at all chemokine receptors in *β*-arrestin recruitment assays. Effect of CXCL14 (1 µM) in *β*-arrestin recruitment assays at all chemokine receptors (CCR1, CCR2, CCR3, CCR4, CCR5, CCR6, CCR7, CCR8, CCR9, CCR10, CXCR1, CXCR2, CXCR3, CXCR4, CXCR5, CXCR6, and CX3CR1) versus their corresponding agonist at their determined EC_{80} (see Supplementary Data 2). CXCL14 was preincubated for 30 min, then the standard agonist for the investigated receptor was added, followed by another incubation for 90 min without washing step in between. The chemokine receptor XCR1, coupled to $G\alpha_i$, was tested in cAMP assays. The readout was relative luminescence units (RLU). The percentage of inhibition was calculated using the following formula: % Inhibition = 100% x (1 - (mean RLU of CXCL14- mean RLU of vehicle control) / (mean RLU of EC_{80} control - mean RLU of vehicle control)). For further details see Supplementary Data 2.

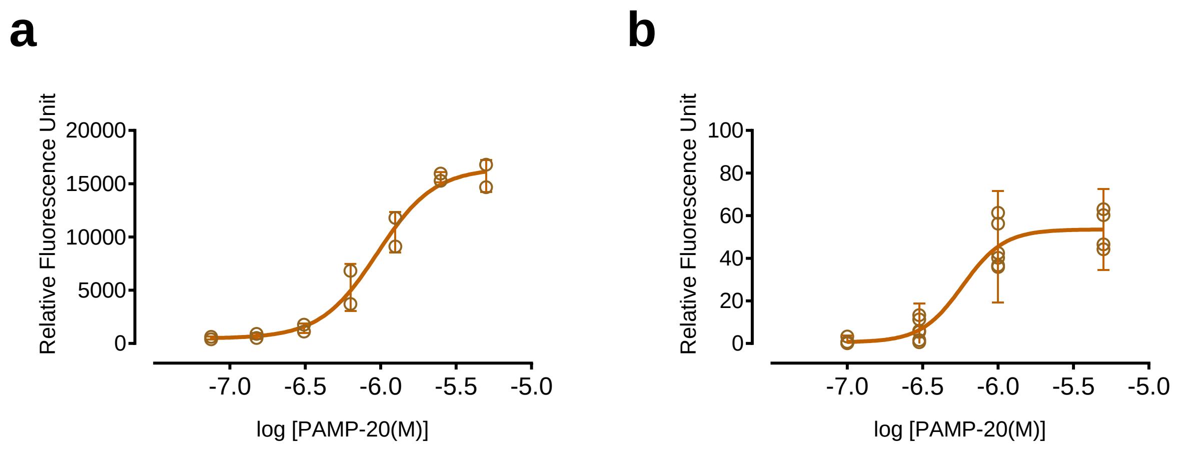

Supplementary Fig. 3: **Control experiments for calcium mobilization assays. a** Concentration-response curve of the MRGPRX2 agonist PAMP-20 in CHEM1 cells recombinantly expressing MRGPRX2 (EC_{50} 0.889 ± 0.163 µM). **b** Concentration-response curve of PAMP-20 in LN229 cells recombinantly expressing MRGPRX2 (EC_{50} 0.950 ± 0.451 µM).

>CXCL1
MARAALSAAPSNPRLLRVALLLLLLVAAGRRAAGASVATELRCQCLQTLQGIHPKNIQSVNVKSPGPHCAQTEVIATLKNG
RKACLNPASPIVKKIIEKMLNSDKSN
>CXCL2
MARATLSAAPSNPRLLRVALLLLLLVAASRRAAGAPLATELRCQCLQTLQGIHLKNIQSVKVKSPGPHCAQTEVIATLKNGQKACLN
PASPMVKKIIEKMLKNGKSN
>CXCL3
MAHATLSAAPSNPRLLRVALLLLLLVAASRRAAGASVVTELRCQCLQTLQGIHLKNIQSVNVRSPGPHCAQTEVIATLKNGKKACLN
PASPMVQKIIEKILNKGSTN
>CXCL4
MSSAAGFCASRPGLLFLGLLLLPLVVAFASAEAEEDGDLQCLCVKTTSQVRPRHITSLEVIKAGPHCPTAQLIATLKNGRKICLDLQ
APLYKKIIKKLLES
>CXCL5
MSLLSSRAARVPGPSSSLCALLVLLLLLTQPGPIASAGPAAAVLRELRCVCLQTTQGVHPKMISNLQVFAIGPQCSKVEVVASLKNG
KEICLDPEAPFLKKVIQKILDGGNKEN
>CXCL6
MSLPSSRAARVPGPSGSLCALLALLLLLTPPGPLASAGPVSAVLTELRCTCLRVTLRVNPKTIGKLQVFPAGPQCSKVEVVASLKNG
KQVCLDPEAPFLKKVIQKILDSGNKKN
>CXCL7
MSLRLDTTPSCNSARPLHALQVLLLLSLLLTALASSTKGQTKRNLAKGKEESLDSDLYAELRCMCIKTTSGIHPKNIQSLEVIGKGT
HCNQVEVIATLKDGRKICLDPDAPRIKKIVQKKLAGDESAD
>CXCL8
MTSKLAVALLAAFLISAALCEGAVLPRSAKELRCQCIKTYSKPFHPKFIKELRVIESGPHCANTEIIVKLSDGRELCLDPKENWVQR
VVEKFLKRAENS
>CXCL9
MKKSGVLFLLGIILLVLIGVQGTPVVRKGRCSCISTNQGTIHLQSLKDLKQFAPSPSCEKIEIIATLKNGVQTCLNPDSADVKELIK
KWEKQVSQKKKQKNGKKHQKKKVLKVRKSQRSRQKKTT
>CXCL10
MNQTAILICCLIFLTLSGIQGVPLSRTVRCTCISISNQPVNPRSLEKLEIIPASQFCPRVEIIATMKKKGEKRCLNPESKAIKNLLK
AVSKERSKRSP
>CXCL11
MSVKGMAIALAVILCATVVQGFPMFKRGRCLCIGPGVKAVKVADIEKASIMYPSNNCDKIEVIITLKENKGQRCLNPKSKQARLIIK
KVERKNF
>CXCL12
MNAKVVVVLVLVLTALCLSDGKPVSLSYRCPCRFFESHVARANVKHLKILNTPNCALQIVARLKNNNRQVCIDPKLKWIQEYLEKAL
NKRFKM
>CXCL13
MKFISTSLLLMLLVSSLSPVQGVLEVYYTSLRCRCVQESSVFIPRRFIDRIQILPRGNGCPRKEIIVWKKNKSIVCVDPQAEWIQRM
MEVLRKRSSSTLPVPVFKRKIP
>CXCL14(mature)
SKCKCSRKGPKIRYSDVKKLEMKPKYPHCEEKMVIITTKSVSRYRGQEHCLHPKLQSTKRFIKWYNAWNEKRRVYEE
>CXCL16
MNRLRKHLRAYHRCLYYTRFQLLSWSVCGGNKDPWVQELMSCLDLKECGHAYSGIVAHQKHLLPTSPPISQASEGASSDIHTPAQML
LSTLQS
>CXCL17
MKVLISSLLLLLPLMLMSMVSSSLNPGVARGHRDRGQASRRWLQEGGQECECKDWFLRAPRRKFMTVSGLPKKQCPCDHFKGNVKKT
RHQRHHRKPNKHSRACQQFLKQCQLRSFALPL

```
CLUSTAL O(1.2.4) multiple sequence alignment


CXCL1       --CQCLQTLQG-IHP--KNIQSVNV-KSPGPHCA---QTEVIATLK-----NGRKACLNP    46
CXCL2       --CQCLQTLQG-IHL--KNIQSVKV-KSPGPHCA---QTEVIATLK-----NGQKACLNP    46
CXCL3       --CQCLQTLQG-IHL--KNIQSVNV-RSPGPHCA---QTEVIATLK-----NGKKACLNP    46
CXCL4       --CLCVKTTSQ-VRP--RHITSLEV-IKAGPHCP---TAQLIATLK-----NGRKICLDL    46
CXCL5       --CVCLQTTQG-VHP--KMISNLQV-FAIGPQCS---KVEVVASLK-----NGKEICLDP    46
CXCL6       --CTCLRVTLR-VNP--KTIGKLQV-FPAGPQCS---KVEVVASLK-----NGKQVCLDP    46
CXCL7       --CMCIKTTSG-IHP--KNIQSLEV-IGKGTHCN---QVEVIATLK-----DGRKICLDP    46
CXCL8       --CQCIKTYSKPFHP--KFIKELRV-IESGPHCA---NTEIIVKLS-----DGRELCLDP    47
CXCL9       --CSCISTNQGTIHL--QSLKDLKQ-FAPSPSCE---KIEIIATLK-----NGVQTCLNP    47
CXCL10      --CTCISISNQPVNP--RSLEKLEI-IPASQFCP---RVEIIATMK----KKGEKRCLNP    48
CXCL11      --CLCIGPGVKAVKV--ADIEKASI-MYPSNNCD---KIEVIITLK----ENKGQRCLNP    48
CXCL12      --CPCRFFESH-VAR--ANVKHLKI-L-NTPNCA---L-QIVARLK----NNNRQVCIDP    45
CXCL13      --CRCVQESSVFIPR--RFIDRIQI-LPRGNGCP---RKEIIVWKK-----NKSIVCVDP    47
CXCL14      SKCKCSRKGPK-IRY--SDVKKLEM-KPKYPHCE---EKMVIITTKSVSRYRGQEHCLHP    53
CXCL16      --CYCGKRISSDSPPSVQFMNRLRKHLRAYHRCLYYTRFQLL----------SWSVCGGN    48
CXCL17      --CECKDWFLRAPRR--KFMTVSGL---PKKQCPC---DHFKGNVKKTR-HQRHHRKPNK    49


CXCL1       ASPIVKKIIEKMLNSDKSN-----------------------------  65
CXCL2       ASPMVKKIIEKMLKNGKSN-----------------------------  65
CXCL3       ASPMVQKIIEKILNKGSTN-----------------------------  65
CXCL4       QAPLYKKIIKKLLES---------------------------------  61
CXCL5       EAPFLKKVIQKILDGGNKEN----------------------------  66
CXCL6       EAPFLKKVIQKILDSGNKKN----------------------------  66
CXCL7       DAPRIKKIVQKKLAGDESAD----------------------------  66
CXCL8       KENWVQRVVEKFLKRAENS-----------------------------  66
CXCL9       DSADVKELIKKWEKQVSQKKKQKNGKKHQKKKVLKVRKSQRSRQKKTT  95
CXCL10      ESKAIKNLLKAVSKERSKRSP---------------------------  69
CXCL11      KSKQARLIIKKVERKNF-------------------------------  65
CXCL12      KLKWIQEYLEKALNKRFKM-----------------------------  64
CXCL13      QAEWIQRMMEVLRKRSSSTLPVPVFKRKIP------------------  77
CXCL14      KLQSTKRFIKWYNAWNEKRRVYE---E---------------------  77
CXCL16      KDPWVQELMSCLDLKECG-HAYSGIVAHQ-------------------  76
CXCL17      HSRACQQFLKQCQLRSFAL-PL--------------------------  70
```

Supplementary Fig. 4: **Sequence alignment of CXCLs.** CXCLs are shown on the top. Partial sequences that might activate MRGPRX2 are highlighted in turquoise. Acidic amino acids near the suggested activating motif are depicted in red; CXCLs that might activate MRGPRX2 are highlighted in yellow; CXCLs that activate MRGPRX2 are highlighted in green. The sequences of CXCLs, except CXCL14, are starting from the CXC-motif to simplify the alignment. The sequence of CXCL16 is reduced to simplify the alignment display.

CXCL14

```
        10         20         30         40         50         60
SKCKCSRKGP KIRYSDVKKL EMKPKYPHCE EKMVIITTKS VSRYRGQEHC LHPKLQSTKR

        70
FIKWYNAWNE KRRVYEE
```

Enzymes predicted to cleave the CXCL14 sequence

Enzyme	Number of cleavages	Positions of cleavage site(s)
**Human proteases**		
Asp-N endopeptidase	1	15
Asp-N endopeptidase + N-terminal Glu	8	15 20 29 30 47 69 75 76
Chymotrypsin-high specificity (C-term to [FYW], not before P)	7	14 44 61 64 65 68 75
Chymotrypsin-low specificity (C-term to [FYWML], not before P)	14	14 20 22 28 33 44 49 51 55 61 64 65 68 75
Glutamyl endopeptidase	7	21 30 31 48 70 76 77
Neutrophil elastase	5	17 34 41 67 74
LysC	14	2 4 8 11 18 19 23 25 32 39 54 59 63 71
LysN	14	1 3 7 10 17 18 22 24 31 38 53 58 62 70
Pepsin (pH 1.3)	3	19 50 55
Pepsin (pH >2)	10	14 19 26 44 50 55 63 64 67 68
Proline-endopeptidase [*]	2	24 53
Proteinase K	30	12 14 17 20 21 26 30 31 34 35 36 37 38 41 44 48 51 55 58 61 62 64 65 67 68 70 74 75 76 77
Trypsin	20	2 4 7 8 11 13 18 19 25 32 39 43 45 54 59 60 63 71 72 73
**Bacterial proteases**		
Arg-C proteinase	7	7 13 43 45 60 72 73
Thermolysin	13	11 19 32 33 34 35 40 50 54 60 61 66 73
Clostripain	7	7 13 43 45 60 72 73
Staphylococcal peptidase I	5	21 30 48 70 76

Enzymes not predicted to cut CXCL14: Caspase1-10 and Enterokinase

- The cleavage occurs at the **right side** (C-terminal direction) of the marked amino acids

```
                                                    ArgC_Therm_Tryps
                                                              Tryps|
                                                             ProtK||
                                          Ch_lo_Pn1.3_Pn2_ProtK  |||
                                                   Therm_Tryps|  |||
                                                Ch_lo_ProtK  ||  |||
                                           Pn1.3_Pn2_Therm|  ||  |||
                                                    Ch_lo||  ||  |||
                                         Glu_ProtK_Staph|||  ||  |||
                                                AspGluN||||  ||  |||
                                           ArgC_Tryps |||||  ||  |||
                               Ch_hi_Ch_lo_Pn2_ProtK| |||||  ||  |||
                                         ArgC_Tryps|| |||||  ||  |||
                                      Elast_ProtK ||| |||||  ||  |||
                                           Therm| ||| |||||  ||  |||
                                          Tryps|| ||| |||||  ||  |||
                                         ProtK||| ||| |||||  ||  |||
                                        ProtK|||| ||| |||||  ||  |||
                                       ProtK||||| ||| |||||  ||  |||
                                ProtK_Therm|||||| ||| |||||  ||  |||
                         Elast_ProtK_Therm||||||| ||| |||||  ||  |||
                              Ch_lo_Therm|||||||| ||| |||||  ||  |||
                             Therm_Tryps||||||||| ||| |||||  ||  |||
                              Glu_ProtK|||||||||| ||| |||||  ||  |||
               AspGluN_Glu_ProtK_Staph||||||||||| ||| |||||  ||  |||
                              AspGluN|||||||||||| ||| |||||  ||  |||
                               Ch_lo||||||||||||| ||| |||||  ||  |||
                         Pn2_ProtK |||||||||||||| ||| |||||  ||  |||
                            Tryps| |||||||||||||| ||| |||||  ||  |||
                         Ch_lo  || |||||||||||||| ||| |||||  ||  |||
              Glu_ProtK_Staph|  || |||||||||||||| ||| |||||  ||  |||
         AspGluN_Ch_lo_ProtK||  || |||||||||||||| ||| |||||  ||  |||
      Pn1.3_Pn2_Therm_Tryps|||  || |||||||||||||| ||| |||||  ||  |||
                     Tryps||||  || |||||||||||||| ||| |||||  ||  |||
              Elast_ProtK|||||  || |||||||||||||| ||| |||||  ||  |||
           AspN_AspGluN ||||||  || |||||||||||||| ||| |||||  ||  |||
 Ch_hi_Ch_lo_Pn2_ProtK| ||||||  || |||||||||||||| ||| |||||  ||  |||
           ArgC_Tryps|| ||||||  || |||||||||||||| ||| |||||  ||  |||
               ProtK||| ||||||  || |||||||||||||| ||| |||||  ||  |||
        Therm_Tryps|||| ||||||  || |||||||||||||| ||| |||||  ||  |||
           Tryps  ||||| ||||||  || |||||||||||||| ||| |||||  ||  |||
     ArgC_Tryps|  ||||| ||||||  || |||||||||||||| ||| |||||  ||  |||
       Tryps  ||  ||||| ||||||  || |||||||||||||| ||| |||||  ||  |||
     Tryps |  ||  ||||| ||||||  || |||||||||||||| ||| |||||  ||  |||

         | |  ||  ||||| ||||||  || |||||||||||||| ||| |||||  ||  |||
        SKCKCSRKGPKIRYSDVKKLEMKPKYPHCEEKMVIITTKSVSRYRGQEHCLHPKLQSTKR
    1   ---------+---------+---------+---------+---------+---------+   60
```

```
               AspGluN_Glu_ProtK_Staph
            AspGluN_Ch_hi_Ch_lo_ProtK|
                         Elast_ProtK||
                   ArgC_Therm_Tryps|||
                        ArgC_Tryps||||
                            Tryps|||||
                 Glu_ProtK_Staph||||||
                        AspGluN|||||||
         Ch_hi_Ch_lo_Pn2_ProtK||||||||
              Elast_Pn2_ProtK|||||||||
                       Therm||||||||||
          Ch_hi_Ch_lo_ProtK|||||||||||
     Ch_hi_Ch_lo_Pn2_ProtK||||||||||||
                Pn2_Tryps|||||||||||||
                   ProtK||||||||||||||
Ch_hi_Ch_lo_ProtK_Therm|||||||||||||||

                      ||||||||||||||||
                      FIKWYNAWNEKRRVYEE
                 61   ---------+-------   77
```

Supplementary Fig. 5: **Predicted cleavage sites for different proteases in the CXCL14 sequence**. The full sequence of CXCL14 (77 amino acids) was investigated using the Expasy PeptideCutter tool (operated by the SIB Swiss Institute of Bioinformatics).

a CXCL14(57-63)

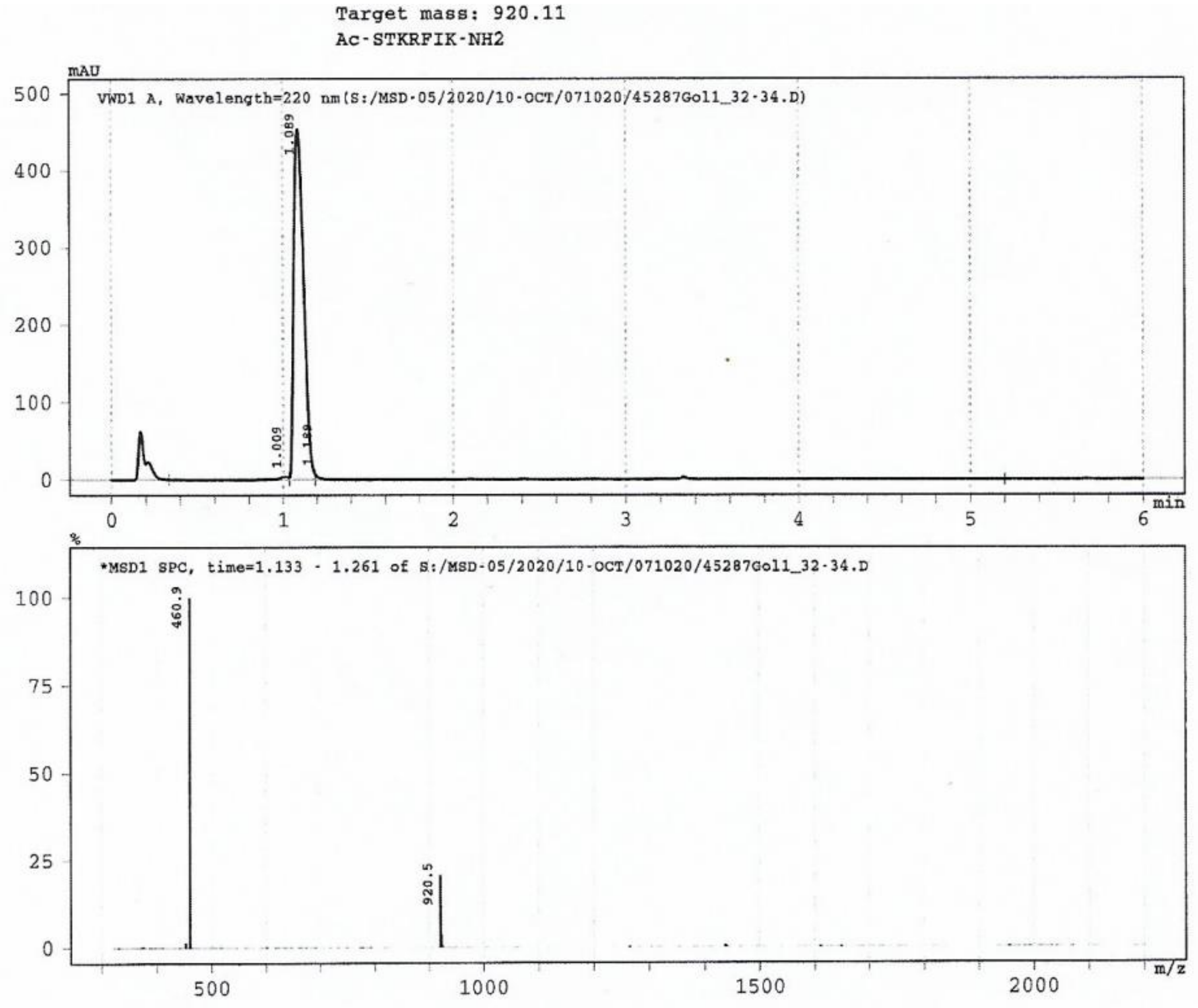

Signal 1: VWD1 A, Wavelength=220 nm

Peak #	RT [min]	Width [min]	Area	Area %
1	1.009	0.70	16.7	0.9
2	1.089	0.15	1844.6	97.5
3	1.189	4.01	30.0	1.6

Supplementary Fig. 6 a: HPLC-UV chromatogram (detection at 220 nm), purity, and mass spectrum of CXCL14(61-65).

b CXCL14(55-65)

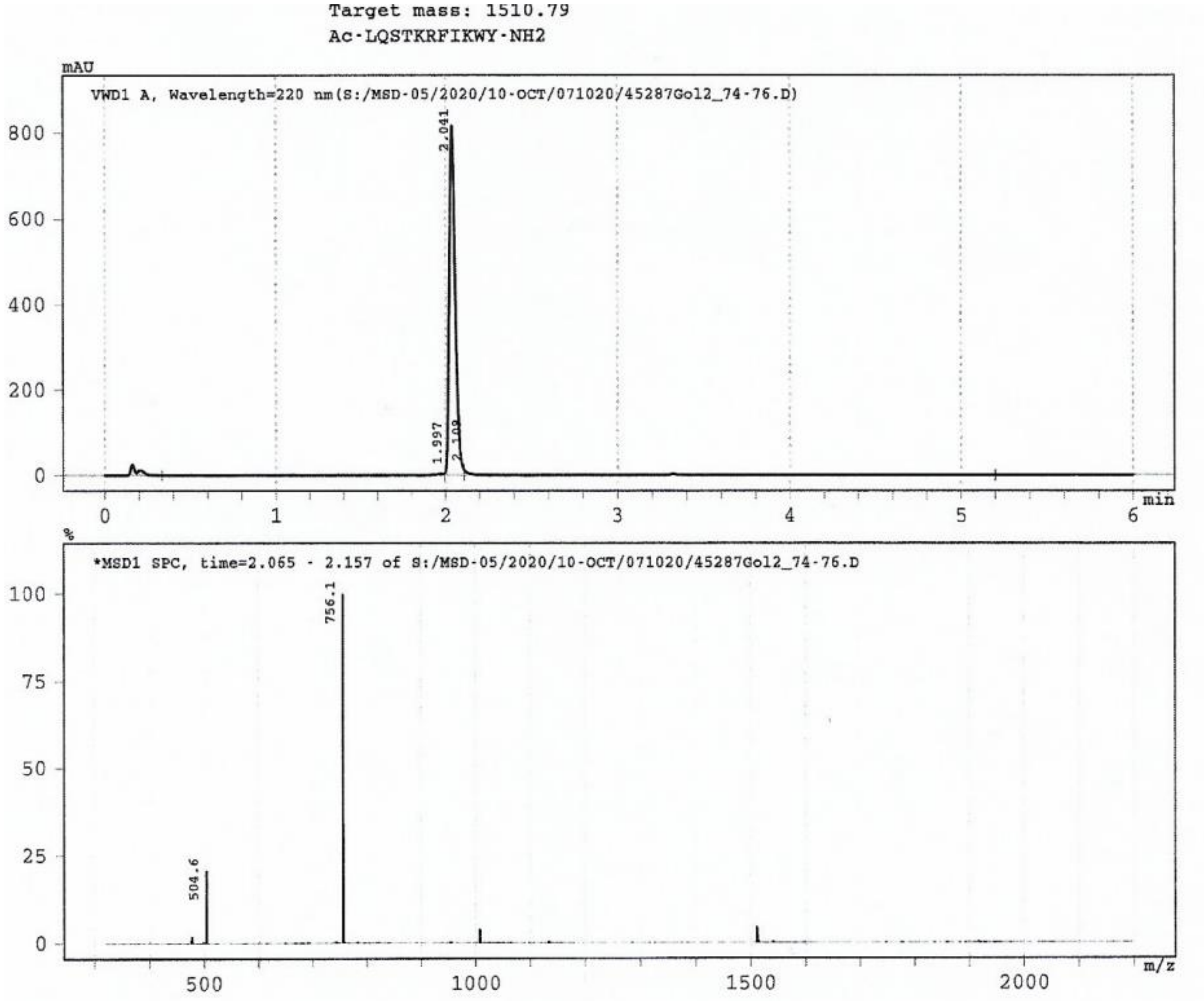

Signal 1: VWD1 A, Wavelength=220 nm

Peak #	RT [min]	Width [min]	Area	Area %
1	1.997	1.66	16.1	0.8
2	2.041	0.11	1995.9	97.5
3	2.109	3.09	34.4	1.7

Supplementary Fig. 6 b: HPLC-UV chromatogram (detection at 220 nm), purity, and mass spectrum of CXCL14(55-65).

c CXCL14(53-67)

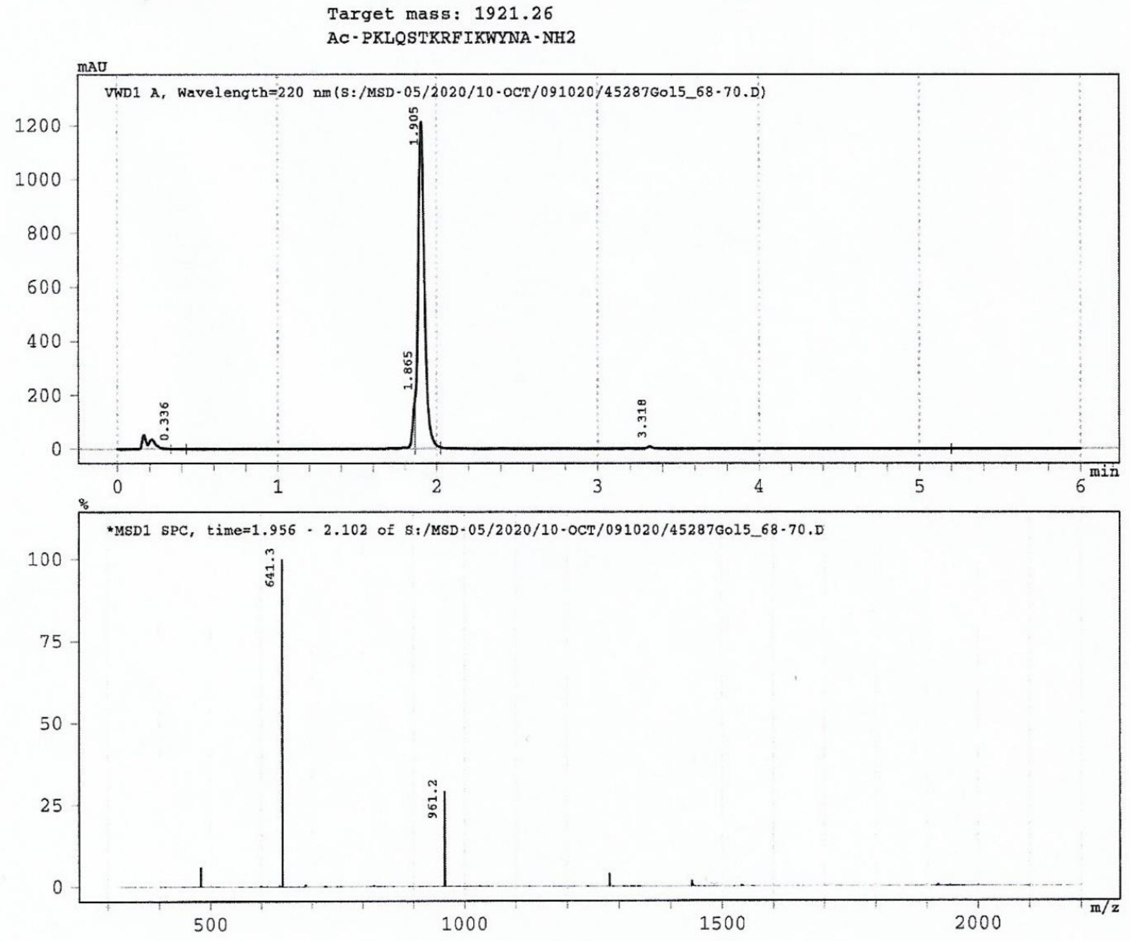

Signal 1: VWD1 A, Wavelength=220 nm

Peak #	RT [min]	Width [min]	Area	Area %
1	0.336	0.09	0.5	0.0
2	1.865	1.44	202.1	5.4
3	1.905	0.16	3470.0	93.3
4	3.318	3.18	45.0	1.2

Supplementary Fig. 6 c: HPLC-UV chromatogram (detection at 220 nm), purity (90.7%), and mass spectrum of CXCL14(53-67).

d CXCL14(53-63)

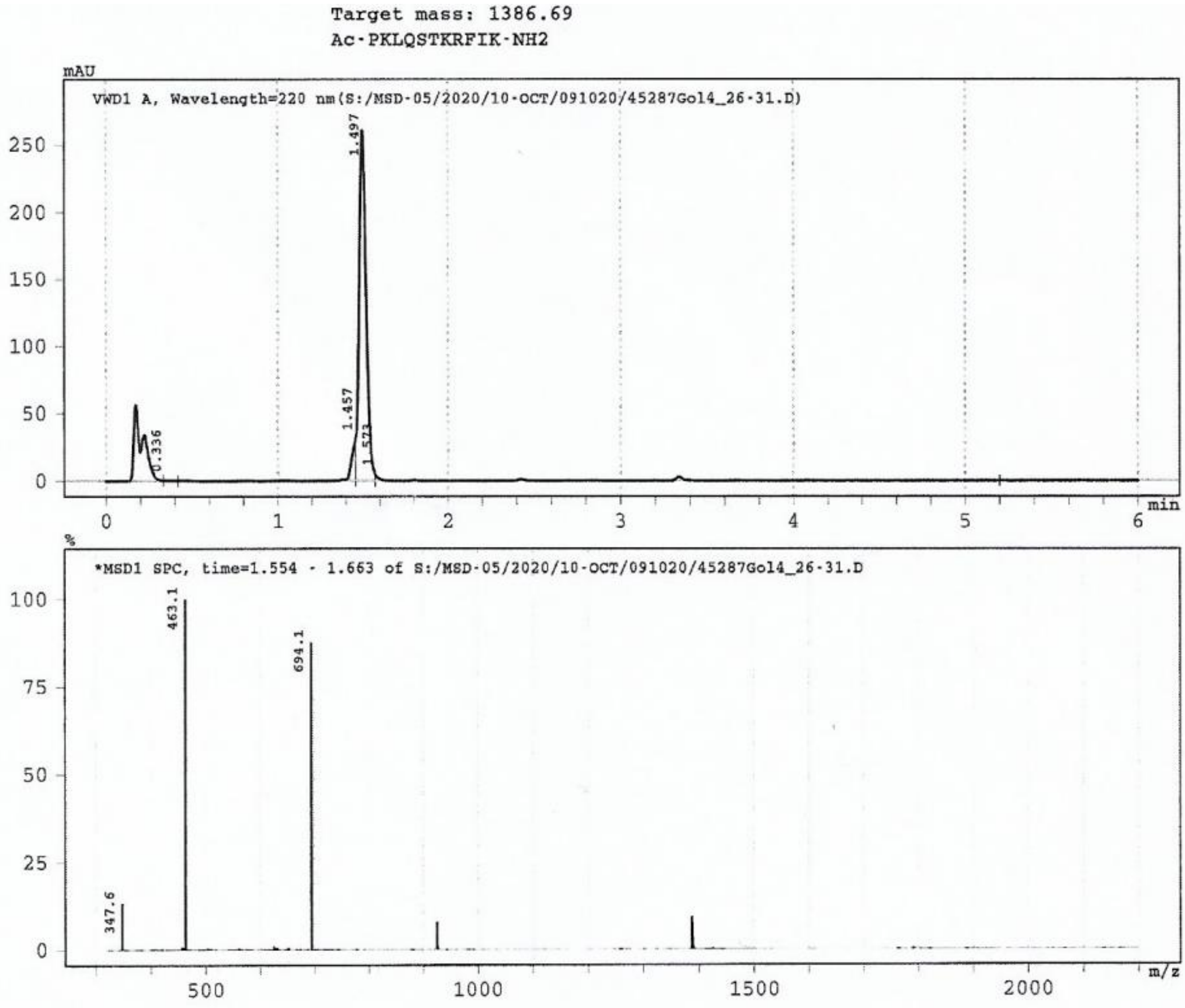

Signal 1: VWD1 A, Wavelength=220 nm

Peak #	RT [min]	Width [min]	Area	Area %
1	0.336	0.08	1.3	0.2
2	1.457	1.04	50.3	6.1
3	1.497	0.12	749.5	90.7
4	1.573	3.63	25.3	3.1

Supplementary Fig. 6 d: HPLC-UV chromatogram (detection at 220 nm), purity, and mass spectrum of CXCL14(53-63).

e CXCL14(57-67)

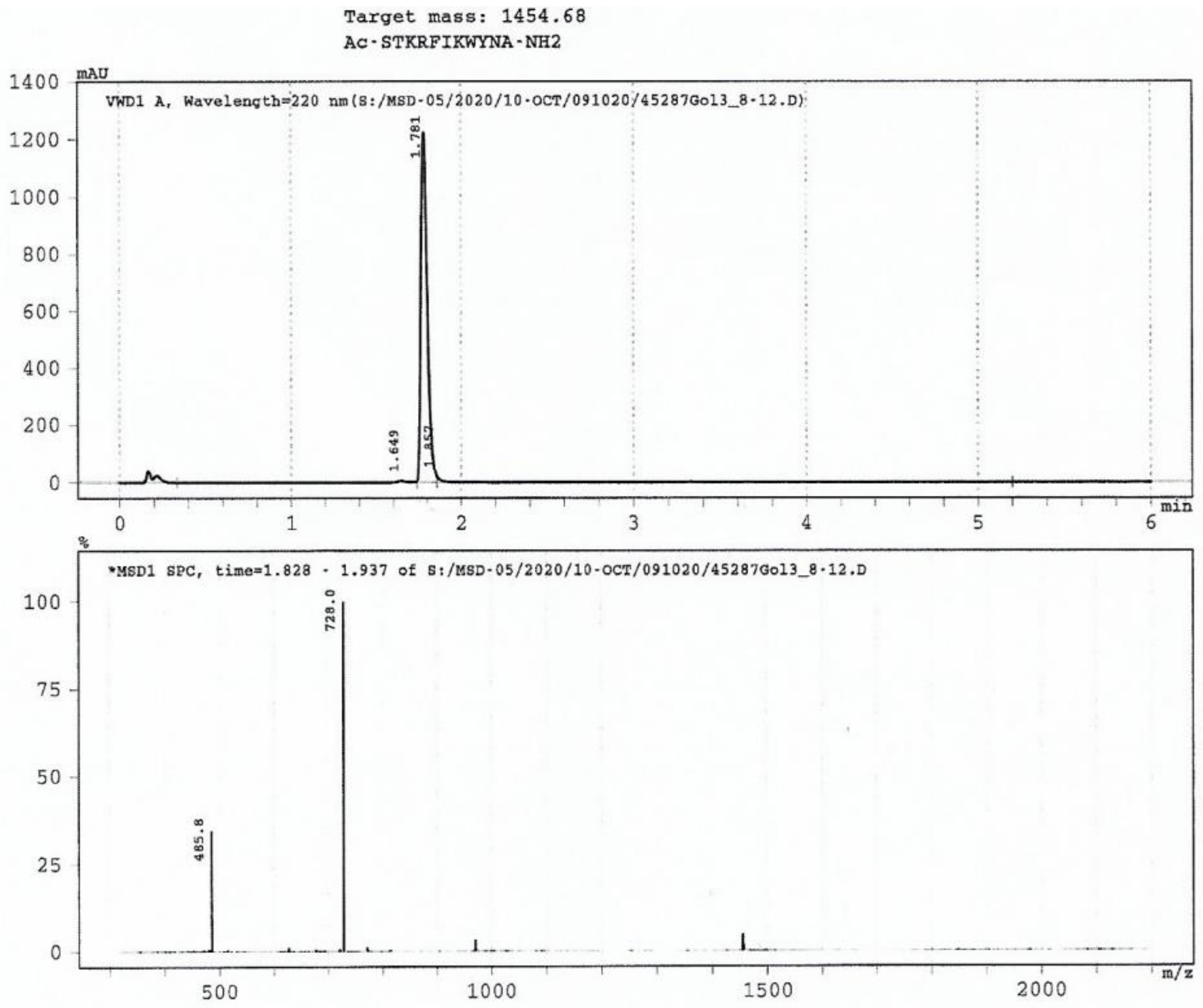

Signal 1: VWD1 A, Wavelength=220 nm

Peak #	RT [min]	Width [min]	Area	Area %
1	1.649	1.41	25.1	0.8
2	1.781	0.12	3178.0	97.8
3	1.857	3.34	47.8	1.5

Supplementary Fig. 6 e: HPLC-UV chromatogram (detection at 220 nm), purity, and mass spectrum of CXCL14(57-67).

f CXCL14(57-65)

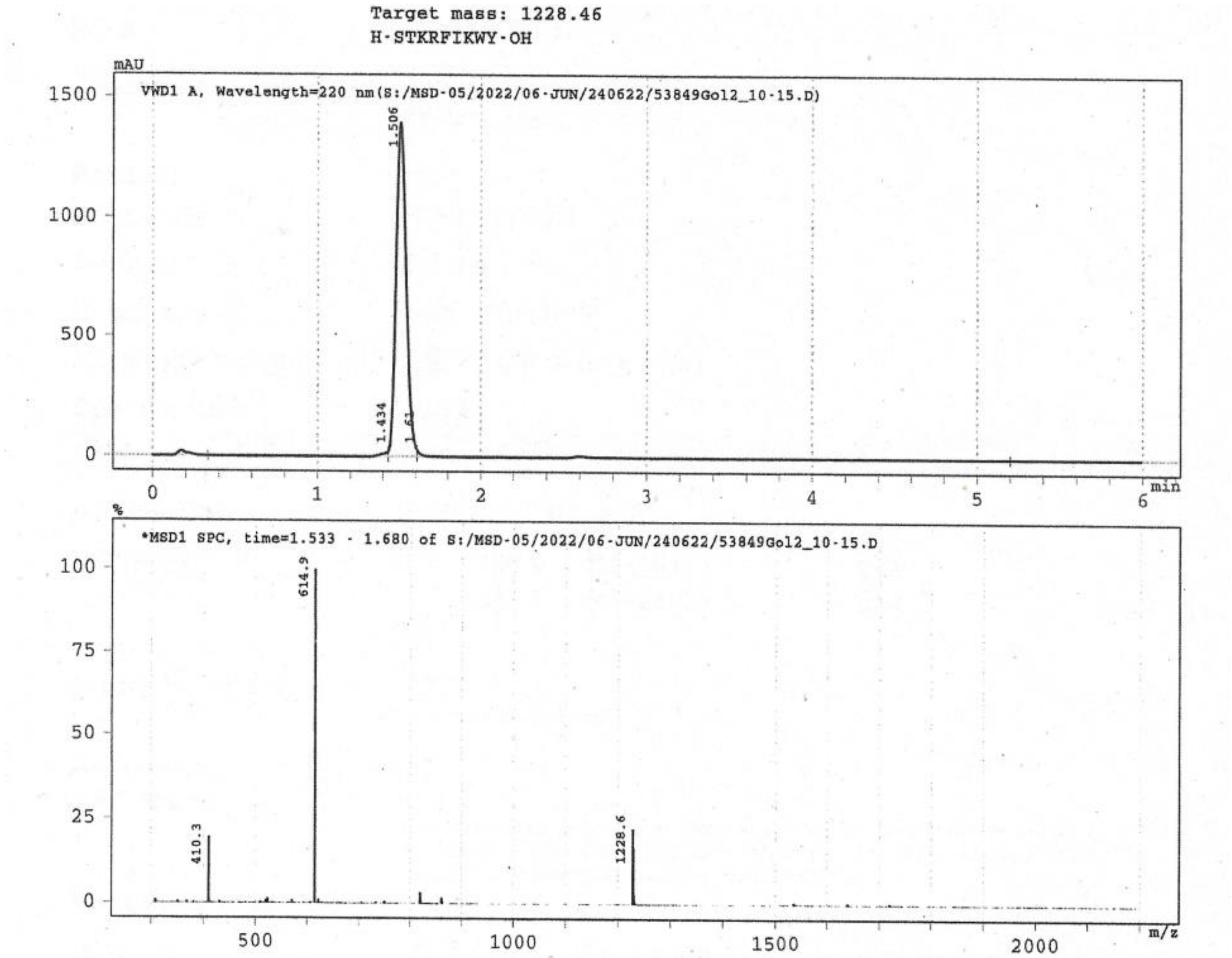

Signal 1: VWD1 A, Wavelength=220 nm

Peak #	RT [min]	Width [min]	Area	Area %
1	1.434	1.10	55.7	0.9
2	1.506	0.18	5982.5	97.5
3	1.610	3.59	99.7	1.6

Supplementary Fig. 6 f: HPLC-UV chromatogram (detection at 220 nm), purity, and mass spectrum of CXCL14(57-65).

g CXCL14(59-65)

Target mass: 1081.32
Ac-KRFIKWY-NH2

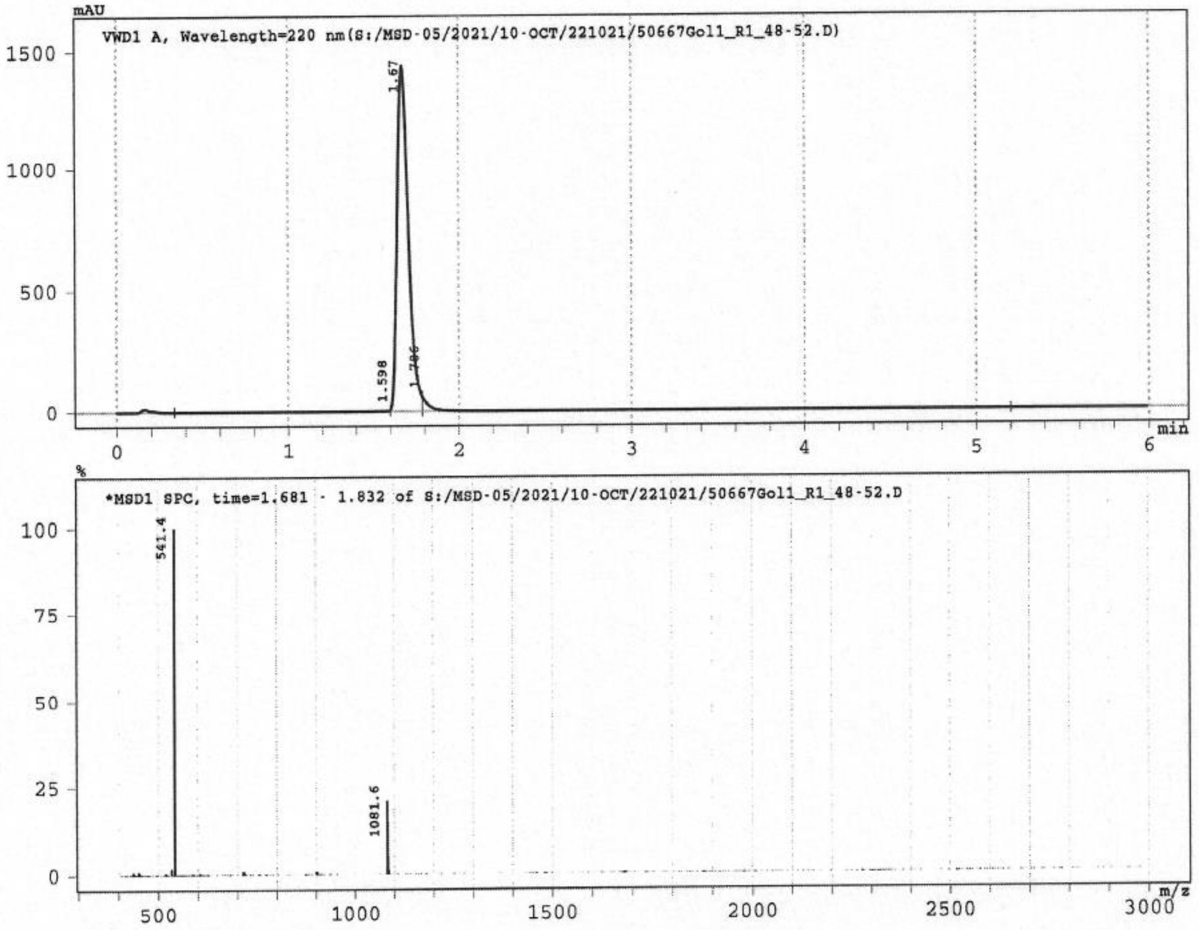

Signal 1: VWD1 A, Wavelength=220 nm

Peak #	RT [min]	Width [min]	Area	Area %
1	1.598	1.26	7.2	0.1
2	1.670	0.19	6553.0	97.3
3	1.786	3.41	175.0	2.6

Supplementary Fig. 6 g: HPLC-UV chromatogram (detection at 220 nm), purity, and mass spectrum of CXCL14(59-65).

h CXCL14(60-65)

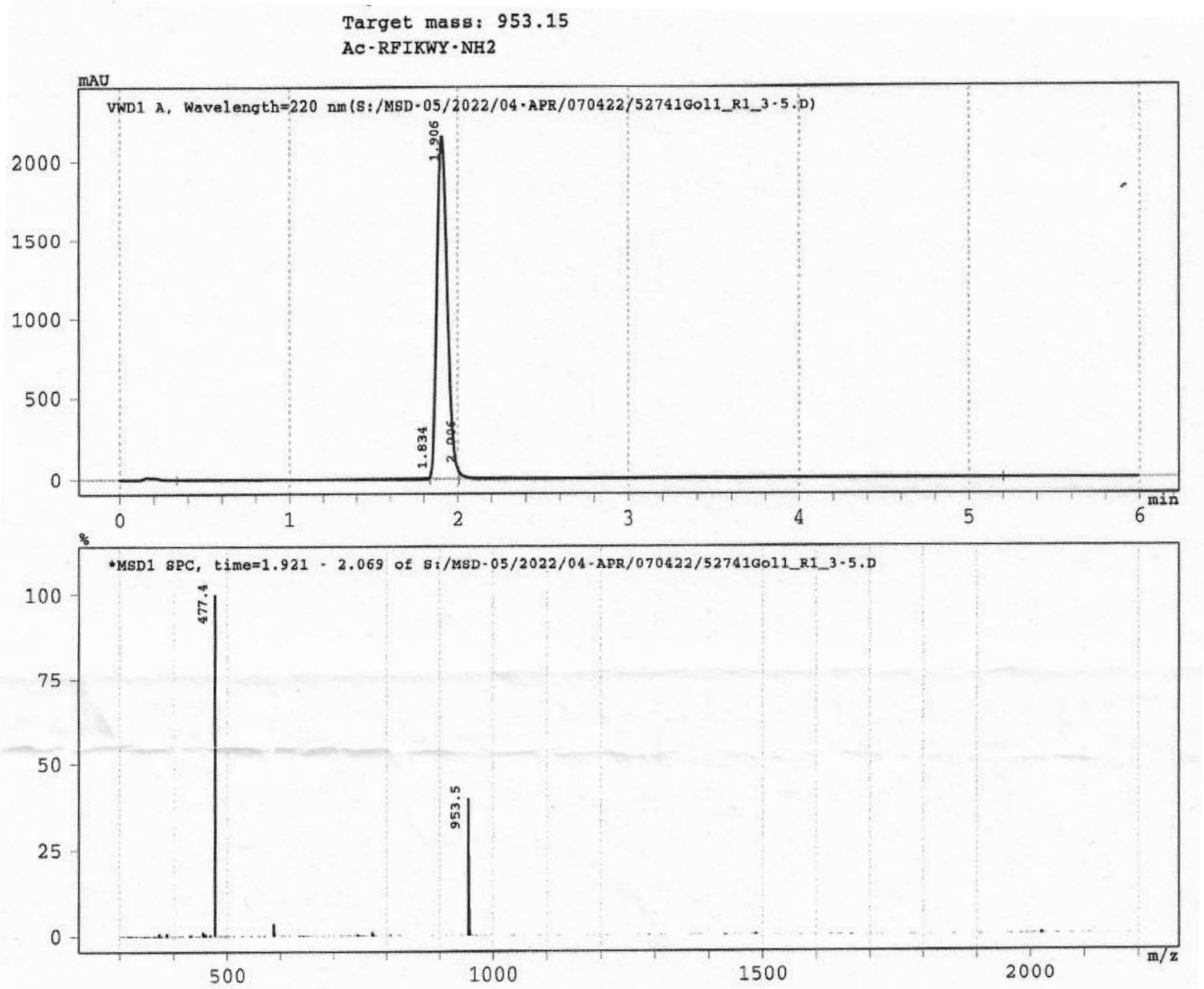

Signal 1: VWD1 A, Wavelength=220 nm

Peak #	RT [min]	Width [min]	Area	Area %
1	1.834	1.50	14.7	0.2
2	1.906	0.17	9081.1	98.8
3	2.006	3.19	99.2	1.1

Supplementary Fig. 6 h: HPLC-UV chromatogram (detection at 220 nm), purity, and mass spectrum of CXCL14(60-65).

i CXCL14(61-65)

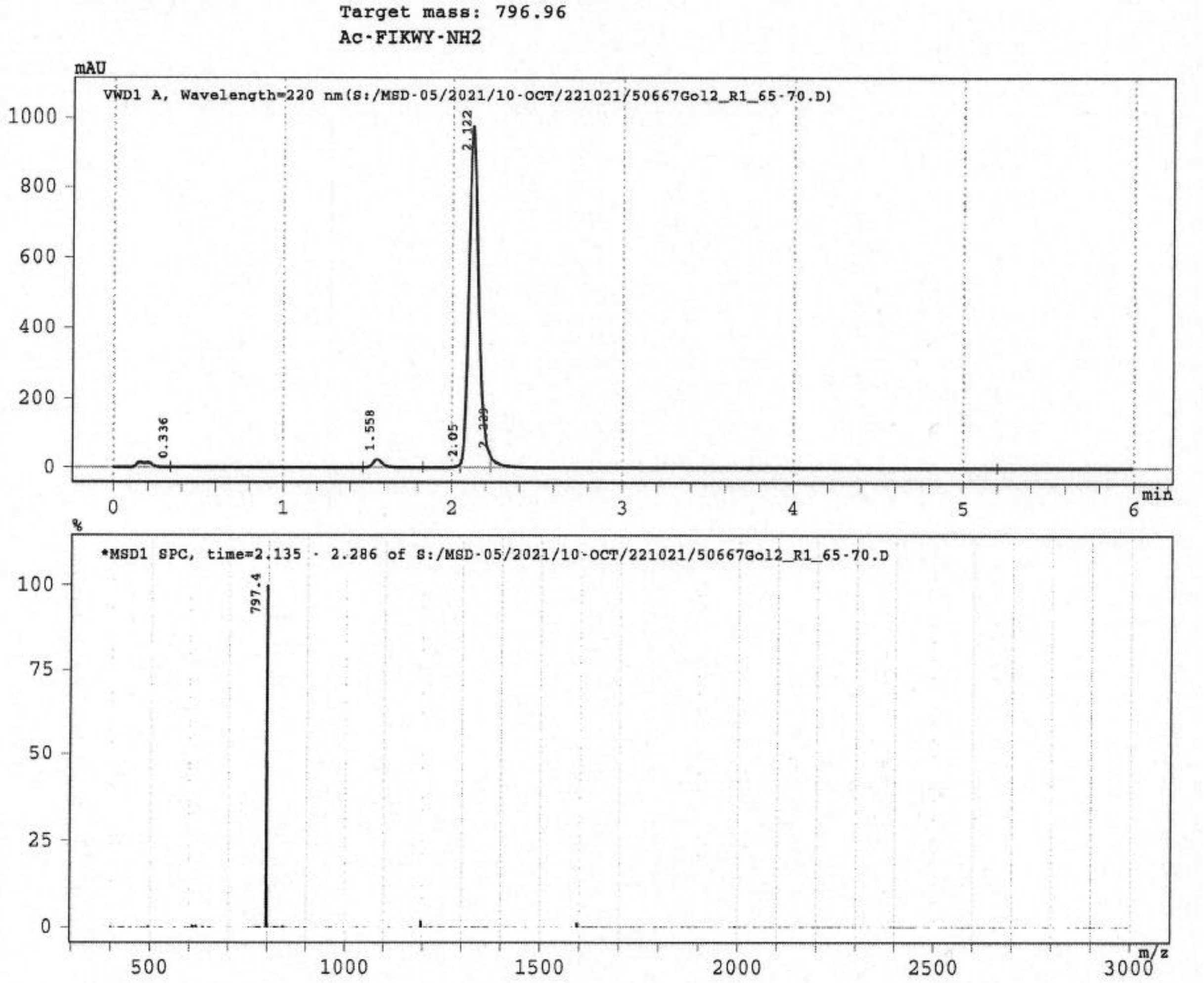

Signal 1: VWD1 A, Wavelength=220 nm

Peak #	RT [min]	Width [min]	Area	Area %
1	0.336	1.14	1.3	0.0
2	1.558	0.36	88.5	2.1
3	2.050	0.22	10.3	0.2
4	2.122	0.18	4021.6	95.5
5	2.229	2.97	90.5	2.1

Supplementary Fig. 6 i: HPLC-UV chromatogram (detection at 220 nm), purity, and mass spectrum of CXCL14(61-65).

j CXCL14(63-65)

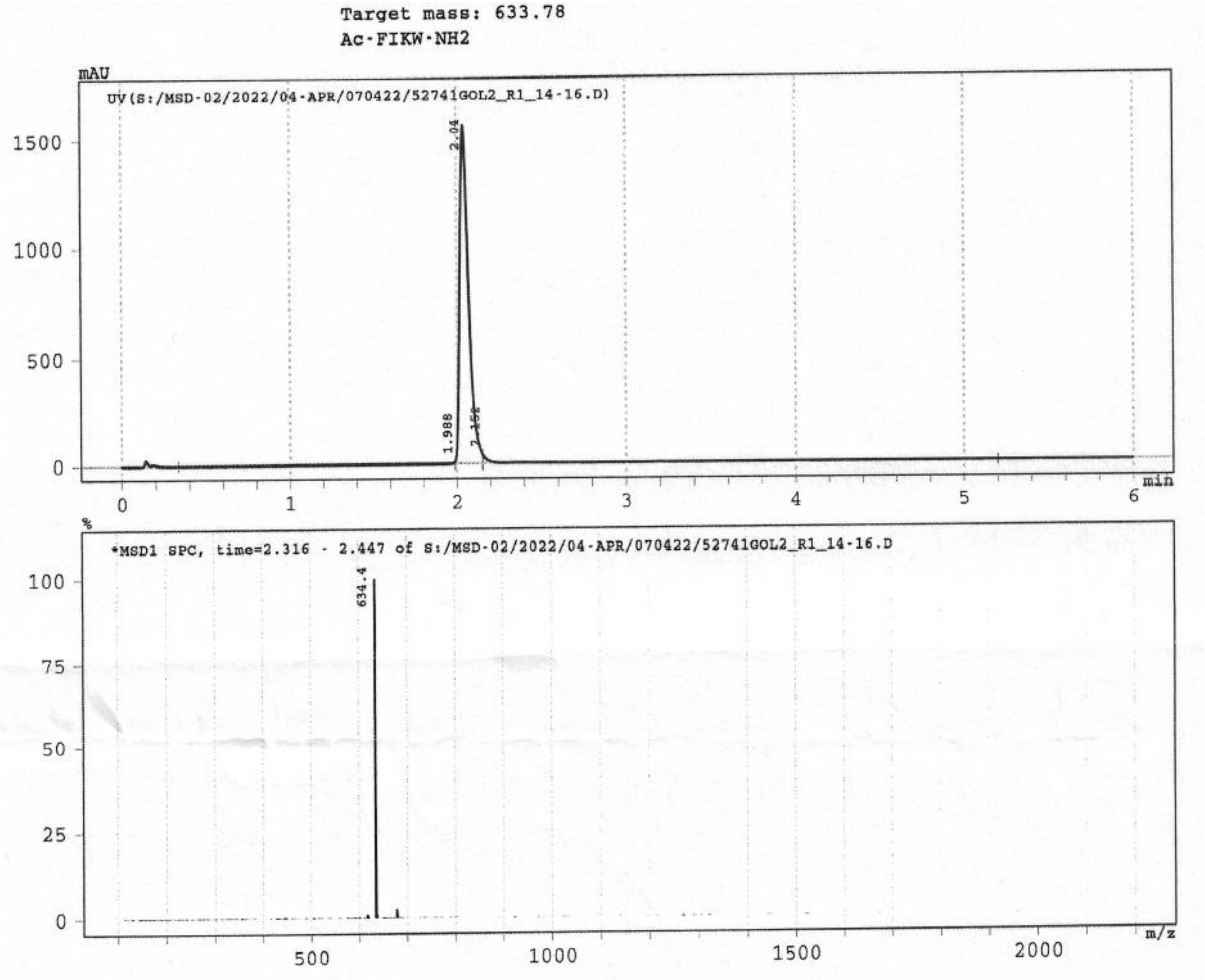

Signal 1: UV

Peak #	RT [min]	Width [min]	Area	Area %
1	1.988	1.65	5.4	0.1
2	2.040	0.16	5733.8	97.8
3	2.152	3.05	123.9	2.1

Supplementary Fig. 6 j: HPLC-UV chromatogram (detection at 220 nm), purity, and mass spectrum of CXCL14(63-65).

k CXCL14(61-64)

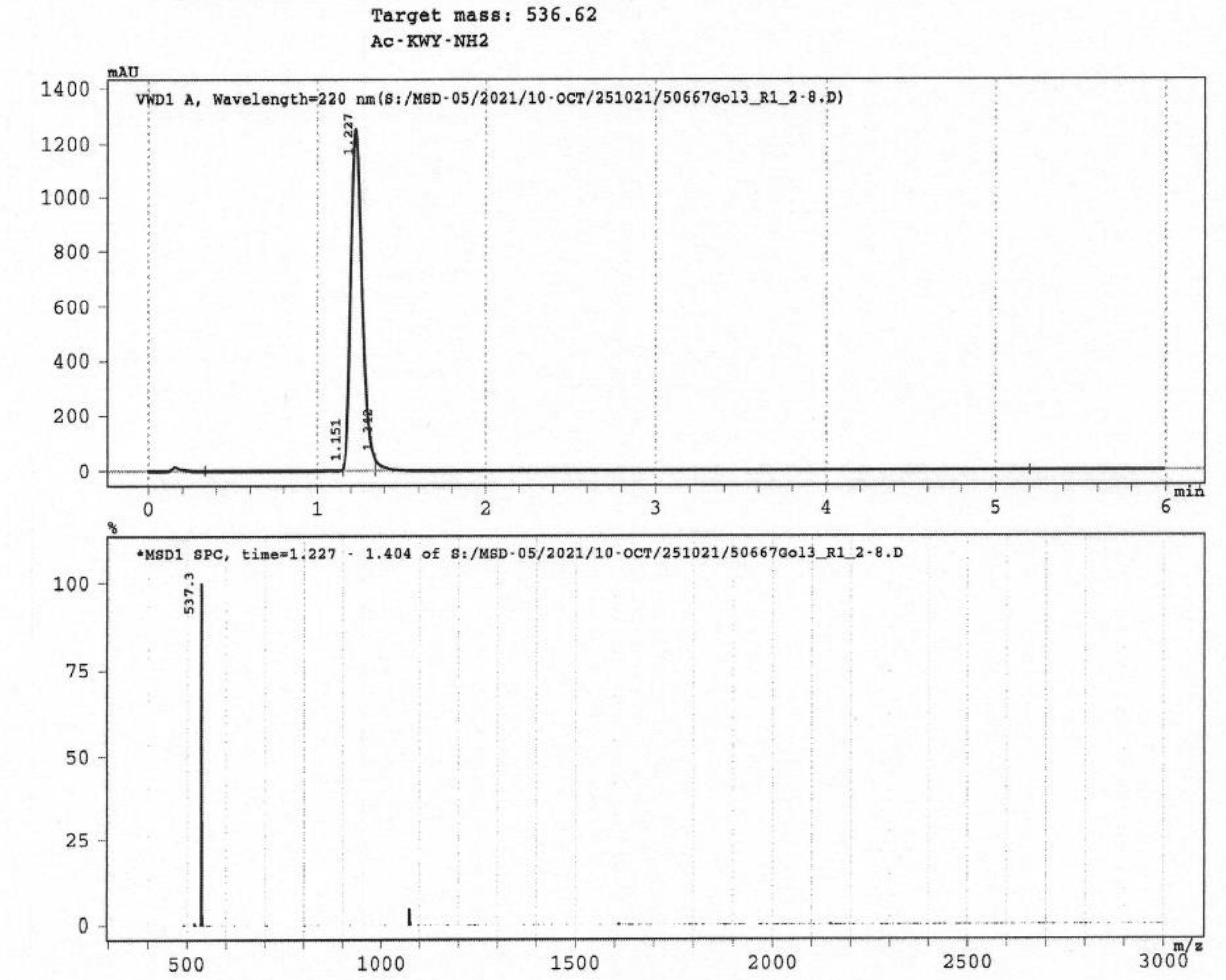

Signal 1: VWD1 A, Wavelength=220 nm

Peak #	RT [min]	Width [min]	Area	Area %
1	1.151	0.82	8.0	0.1
2	1.227	0.19	5832.0	97.7
3	1.342	3.86	126.4	2.1

Supplementary Fig. 6 k: HPLC-UV chromatogram (detection at 220 nm), purity, and mass spectrum of CXCL14(61-64).

l CXCL14(57-65) K59R

Target mass: 1212.41
Ac-STARFIKWY-NH2

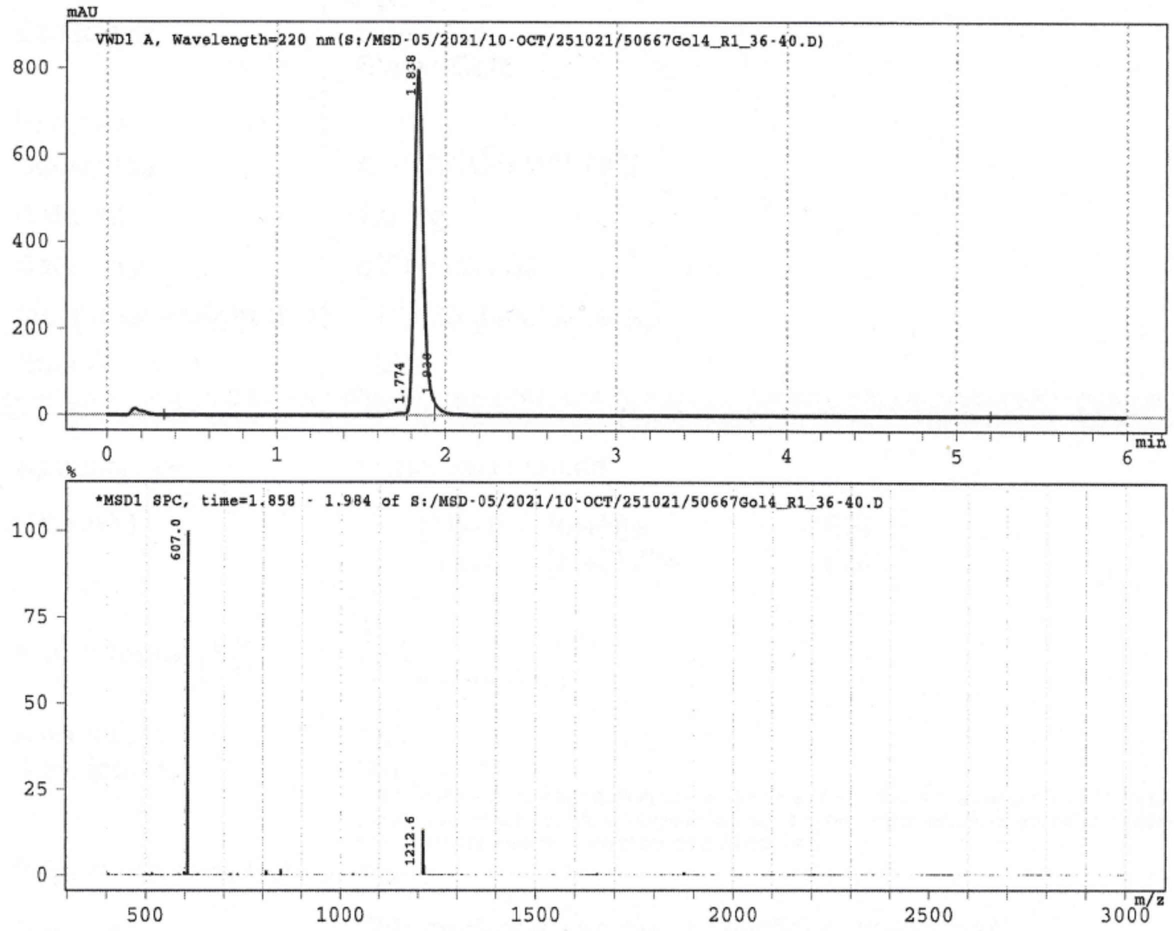

Signal 1: VWD1 A, Wavelength=220 nm

Peak #	RT [min]	Width [min]	Area	Area %
1	1.774	1.44	25.4	0.8
2	1.838	0.16	2927.4	96.5
3	1.938	3.26	81.4	2.7

Supplementary Fig. 6 l: HPLC-UV chromatogram (detection at 220 nm), purity, and mass spectrum of CXCL14(57-65) K59R.

m CXCL14(57-65) K59A

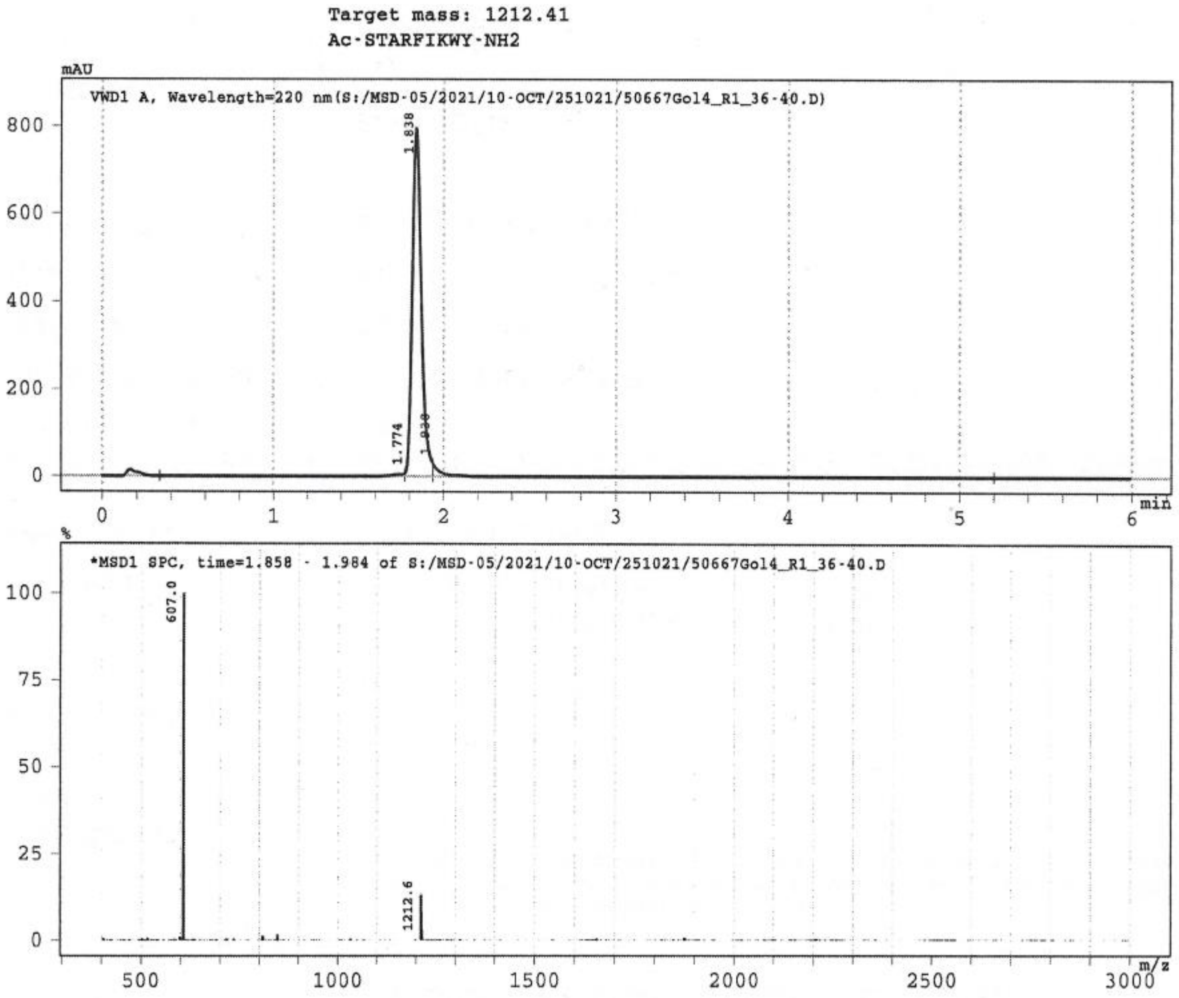

Signal 1: VWD1 A, Wavelength=220 nm

Peak #	RT [min]	Width [min]	Area	Area %
1	1.774	1.44	25.4	0.8
2	1.838	0.16	2927.4	96.5
3	1.938	3.26	81.4	2.7

Supplementary Fig. 6 m: HPLC-UV chromatogram (detection at 220 nm), purity, and mass spectrum of CXCL14(57-65) K59A.

n CXCL14(57-65) R60A

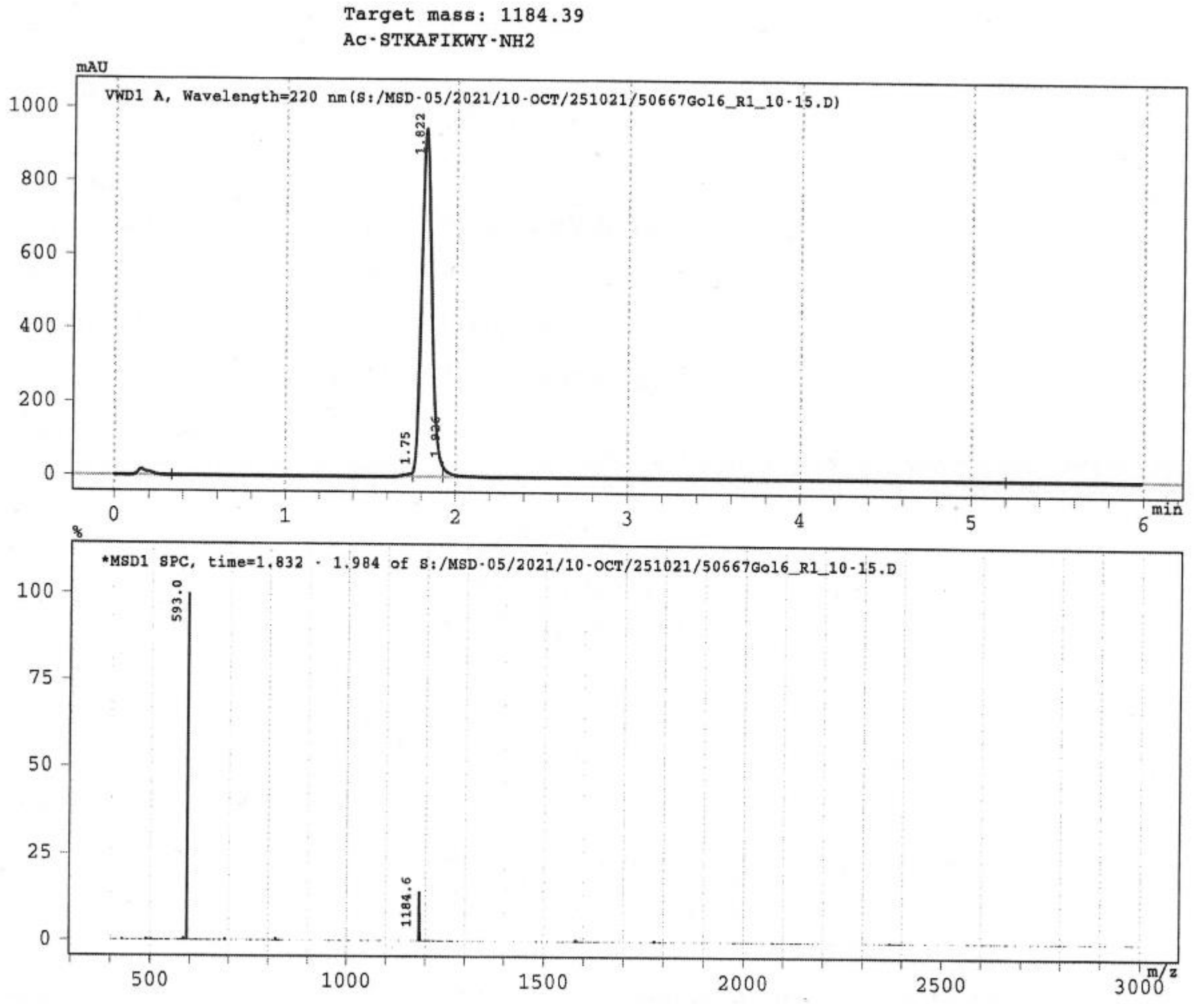

Signal 1: VWD1 A, Wavelength=220 nm

Peak #	RT [min]	Width [min]	Area	Area %
1	1.750	1.42	30.2	0.7
2	1.822	0.18	3945.0	97.5
3	1.926	3.27	69.6	1.7

Supplementary Fig. 6 n: HPLC-UV chromatogram (detection at 220 nm), purity, and mass spectrum of CXCL14(57-65) R60A.

o CXCL14(57-65) K63A

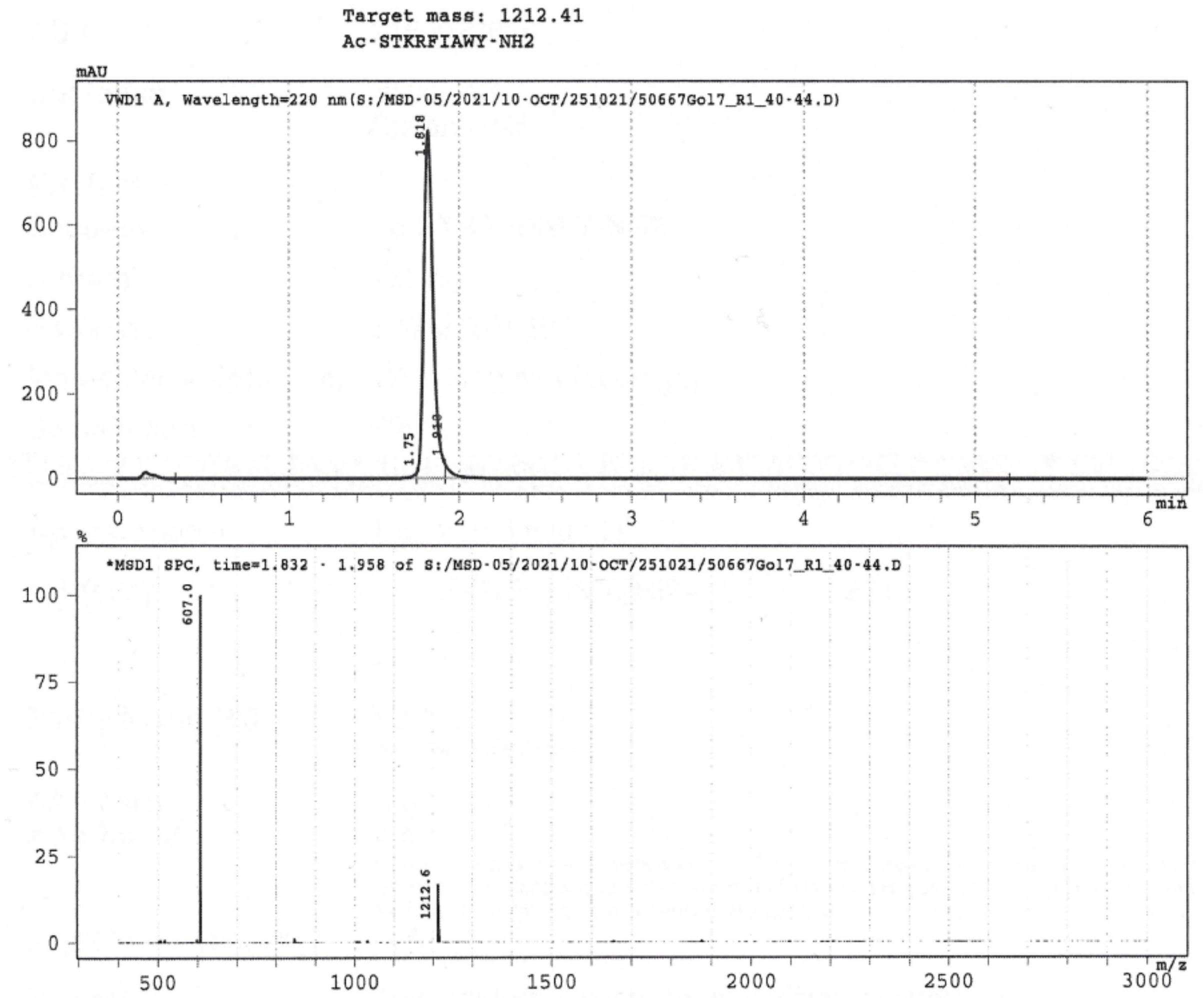

Signal 1: VWD1 A, Wavelength=220 nm

Peak #	RT [min]	Width [min]	Area	Area %
1	1.750	1.42	20.0	0.6
2	1.818	0.17	3151.0	96.5
3	1.918	3.28	93.8	2.9

Supplementary Fig. 6 o: HPLC-UV chromatogram (detection at 220 nm), purity, and mass spectrum of CXCL14(57-65) K63A.

p CXCL14(57-65) W64F

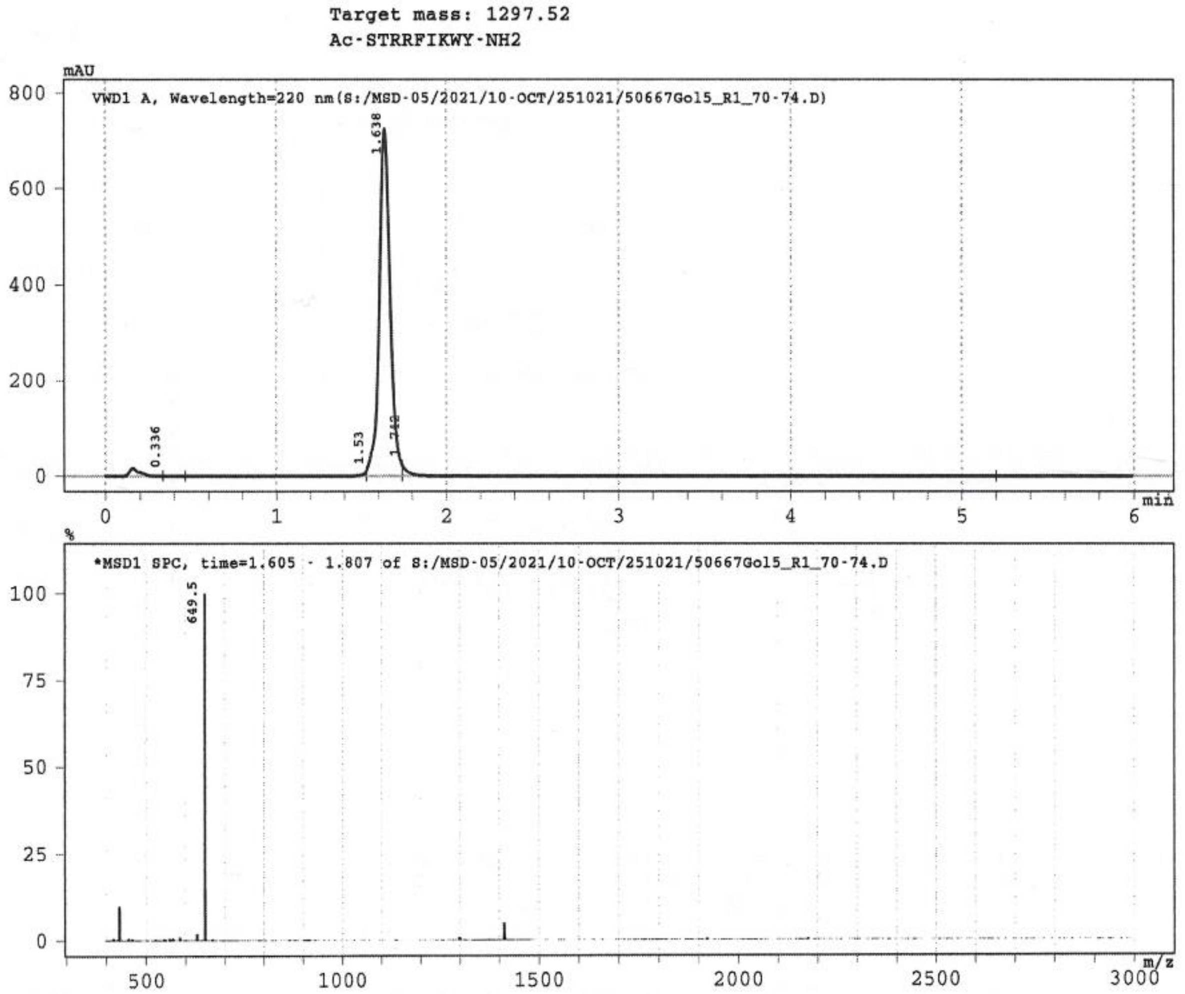

Signal 1: VWD1 A, Wavelength=220 nm

Peak #	RT [min]	Width [min]	Area	Area %
1	0.336	0.13	0.6	0.0
2	1.530	1.07	11.7	0.4
3	1.638	0.21	3221.1	98.1
4	1.742	3.46	50.9	1.5

Supplementary Fig. 6 p: HPLC-UV chromatogram (detection at 220 nm), purity, and mass spectrum of CXCL14(57-65) W64F.

Supplementary Fig. 6: Analytical data of custom-synthesized peptides. The peptides were purified by high performance liquid chromatography and purity was determined by UV detection at 220 nm. Products were further analyzed by liquid chromatography–mass spectrometry for structural confirmation.

Supplementary Table. Bias factor of short CXCL14 peptides:

Ligand	G protein[a]				Arrestin[c]				Ligand bias
	Emax (%)[b]	EC_{50} (nM)	Log ($Emax/EC_{50}$)	ΔLog ($Emax/EC_{50}$)	Emax (%)[b]	EC_{50} (nM)[#]	Log ($Emax/EC_{50}$)	ΔLog ($Emax/EC_{50}$)	Bias factor ΔΔLog ($Emax/EC_{50}$)
**CXCL14**	100	504	6.3	0.0 (ref.)	100	905	6.0	0.0 (ref.)	0.0
**CXCL14 (55-65)**	165	366	6.7	0.4	116	7,770	5.2	-0.9	1.2
**CXCL14 (53-67)**	160	353	6.7	0.4	125	2,820	5.6	-0.4	0.8
**CXCL14 (57-67)**	148	248	6.8	0.5	189	2,700	5.8	-0.2	0.7
**CXCL14 (57-65)**	170	301	6.8	0.5	195	1,980	6.0	0.0	0.5
**CXCL14 (59-65)**	155	316	6.7	0.4	178	1,300	6.1	0.1	0.3
**CXCL14 (60-65)**	130	2,150	5.8	-0.5	133	5,740	5.4	-0.7	0.2
**CXCL14 (61-65)**	128	937	6.1	-0.2	175	3,490	5.7	-0.3	0.2
**CXCL14 (61-64)**	103	3970	5.4	-0.9	117	15,900	4.9	-1.2	0.3

[a] Calcium assays in LN229 cells recombinantly expressing MRGPRX2
[b] Normalized to the maximal effect of CXCL14 1,000 nM (100%)
[c] *β*-Arrestin assays in *β*-Arrestin CHO cells recombinantly expressing MRGPRX2

The bias factor was calculated using the following equation:

$\Delta\Delta\log(E_{max}/EC_{50}) = \Delta\log(E_{max}/EC_{50})$calcium assay - $\Delta\log(E_{max}/EC_{50})$β-arrestin assay, where $\Delta\log(E_{max}/EC_{50}) = \log(E_{max}/EC_{50})$standard agonist - $\log(E_{max}/EC_{50})$peptide.[1] The used standard agonist in this equation is PAMP-20. A bias factor of 0 means no bias, whereas a factor of 1 corresponds to a 10-fold preference of the G-protein pathway over the β-arrestin pathway.

Supplementary References

1. Voss, J. H., Mahardhika, A. B., Inoue, A. & Müller, C. E. Agonist-dependent coupling of the promiscuous adenosine A_{2B} receptor to Gα protein subunits. *ACS Pharmacol. Transl. Sci.* **5,** 373–386; 10.1021/acsptsci.2c00020 (2022).
