# Supplementary material for: Proinflammatory chemokine CXCL14 activates MAS-related G protein-coupled receptor MRGPRX2 and its putative mouse ortholog MRGPRB2
Source: Commun Biol. 2024 Jan 6;7:52. doi: 10.1038/s42003-023-05739-5 (PMC10771525; doi:10.1038/s42003-023-05739-5)
Supplement: Supplementary file 2 — Description of Additional Supplementary Files [file 42003_2023_5739_MOESM2_ESM.pdf]

Description of Additional Supplementary Files

File name: Supplementary Data 1
Description: The data and analysis behind Figure 1a. GEOdata IPF all genes and analysis.

File name: Supplementary Data 2
Description: The data, analysis, and controls behind Figure 1b. GPCR panel screening. CXCL14 was screened at 238 GPCRs as agonist (1 μM) and at 160 GPCRs as antagonist (1 μM); control agonists, EC50 and EC80 values are provided.

File name: Supplementary Data 3
Description: The source data behind the graphs in the paper.
