# Supplementary material for: Proinflammatory chemokine CXCL14 activates MAS-related G protein-coupled receptor MRGPRX2 and its putative mouse ortholog MRGPRB2
Source: Commun Biol. 2024 Jan 6;7:52. doi: 10.1038/s42003-023-05739-5 (PMC10771525; doi:10.1038/s42003-023-05739-5)
Supplement: Supplementary file 6 — Reporting Summary [file 42003_2023_5739_MOESM6_ESM.pdf]

nature portfolio

Corresponding author(s): Christa E. Müller

Last updated by author(s): Oct 16, 2023

Reporting Summary

Nature Portfolio wishes to improve the reproducibility of the work that we publish. This form provides structure for consistency and transparency in reporting. For further information on Nature Portfolio policies, see our Editorial Policies and the Editorial Policy Checklist.

Statistics

For all statistical analyses, confirm that the following items are present in the figure legend, table legend, main text, or Methods section.

n/a	Confirmed	
☐	☒	The exact sample size (*n*) for each experimental group/condition, given as a discrete number and unit of measurement
☐	☒	A statement on whether measurements were taken from distinct samples or whether the same sample was measured repeatedly
☐	☒	The statistical test(s) used AND whether they are one- or two-sided<br>*Only common tests should be described solely by name; describe more complex techniques in the Methods section.*
☐	☒	A description of all covariates tested
☐	☒	A description of any assumptions or corrections, such as tests of normality and adjustment for multiple comparisons
☐	☒	A full description of the statistical parameters including central tendency (e.g. means) or other basic estimates (e.g. regression coefficient) AND variation (e.g. standard deviation) or associated estimates of uncertainty (e.g. confidence intervals)
☐	☒	For null hypothesis testing, the test statistic (e.g. *F*, *t*, *r*) with confidence intervals, effect sizes, degrees of freedom and *P* value noted<br>*Give P values as exact values whenever suitable.*
☒	☐	For Bayesian analysis, information on the choice of priors and Markov chain Monte Carlo settings
☒	☐	For hierarchical and complex designs, identification of the appropriate level for tests and full reporting of outcomes
☒	☐	Estimates of effect sizes (e.g. Cohen's *d*, Pearson's *r*), indicating how they were calculated

Our web collection on statistics for biologists contains articles on many of the points above.

Software and code

Policy information about availability of computer code

Data collection	SoftMax® Pro Software to collect data from FlexStation 3 Multi-Mode Microplate Reader<br>MikroWin 2000 Software to collect data from Mithras Plate Reader LB 940, Berthold Technologies<br>NIS Element Advanced Research Software 4.0 were used for image acquisition and analysis
Data analysis	GraphPad PRISM 8 (GraphPad, San Diego, CA, USA)<br>Molecular Operating Environment (MOE 2018.01)<br>AutoDock4.2.<br>NIS Element Advanced Research software 4.0<br>Online tools:<br>PeptideCutter Expasy SIB Swiss Institute of Bioinformatics (https://web.expasy.org/peptide_cutter/)<br>Gene Expression Omnibus database (https://www.ncbi.nlm.nih.gov/geo/)<br>Sequence Alignment tool ClustaOmega EMBL-EBI (https://www.ebi.ac.uk/Tools/msa/clustalo/)<br>GPCRDB (https://gpcrdb.org/protein/mrgx2_human/)

For manuscripts utilizing custom algorithms or software that are central to the research but not yet described in published literature, software must be made available to editors and reviewers. We strongly encourage code deposition in a community repository (e.g. GitHub). See the Nature Portfolio guidelines for submitting code & software for further information.

Data

Policy information about availability of data

All manuscripts must include a data availability statement. This statement should provide the following information, where applicable:

- Accession codes, unique identifiers, or web links for publicly available datasets
- A description of any restrictions on data availability
- For clinical datasets or third party data, please ensure that the statement adheres to our policy

The data supporting the findings of this study are available in a Source Data file in the Supplementary Information Excel files. Further information is available upon reasonable request from the corresponding author.

Human research participants

Policy information about studies involving human research participants and Sex and Gender in Research.

Reporting on sex and gender	*Use the terms sex (biological attribute) and gender (shaped by social and cultural circumstances) carefully in order to avoid confusing both terms. Indicate if findings apply to only one sex or gender; describe whether sex and gender were considered in study design whether sex and/or gender was determined based on self-reporting or assigned and methods used. Provide in the source data disaggregated sex and gender data where this information has been collected, and consent has been obtained for sharing of individual-level data; provide overall numbers in this Reporting Summary. Please state if this information has not been collected. Report sex- and gender-based analyses where performed, justify reasons for lack of sex- and gender-based analysis.*
Population characteristics	*Describe the covariate-relevant population characteristics of the human research participants (e.g. age, genotypic information, past and current diagnosis and treatment categories). If you filled out the behavioural & social sciences study design questions and have nothing to add here, write "See above."*
Recruitment	*Describe how participants were recruited. Outline any potential self-selection bias or other biases that may be present and how these are likely to impact results.*
Ethics oversight	*Identify the organization(s) that approved the study protocol.*

Note that full information on the approval of the study protocol must also be provided in the manuscript.

Field-specific reporting

Please select the one below that is the best fit for your research. If you are not sure, read the appropriate sections before making your selection.

☒ Life sciences ☐ Behavioural & social sciences ☐ Ecological, evolutionary & environmental sciences

For a reference copy of the document with all sections, see nature.com/documents/nr-reporting-summary-flat.pdf

Life sciences study design

All studies must disclose on these points even when the disclosure is negative.

Sample size	The sample size was chosen that allowed to perform a robust statistical analysis.
Data exclusions	No data were excluded in this study
Replication	All attempts to replicate were successful.
Randomization	Randomization was not appropriate for our assays.
Blinding	Blinding was not appropriate for our assays.

Reporting for specific materials, systems and methods

We require information from authors about some types of materials, experimental systems and methods used in many studies. Here, indicate whether each material, system or method listed is relevant to your study. If you are not sure if a list item applies to your research, read the appropriate section before selecting a response.

Materials & experimental systems

n/a	Involved in the study
☐	☒ Antibodies
☐	☒ Eukaryotic cell lines
☒	☐ Palaeontology and archaeology
☒	☐ Animals and other organisms
☒	☐ Clinical data
☒	☐ Dual use research of concern

Methods

n/a	Involved in the study
☒	☐ ChIP-seq
☒	☐ Flow cytometry
☒	☐ MRI-based neuroimaging

Antibodies

Antibodies used

The mouse monoclonal anti-PK/PL antibody (Catalog #: 92-0010) (DiscoverX, Fremont, CA, USA)
The goat anti-mouse-AlexaFluor488 (Catalog #: 115-545-003) (Jackson Immuno Research, Hamburg, Germany)

Validation

The antibodies were validated by the supplier.

Eukaryotic cell lines

Policy information about cell lines and Sex and Gender in Research

Cell line source(s)

CHEM1 (Catalog #: HTSCHEM-1RTA); CHEM1-MRGPRX2 (Catalog #: HTS058RTA); 1321N1 Astrocytoma cells (Catalog #: 86030402) from Merck-Millipore, Darmstadt, Germany.
CHO-K1-β-arrestin-2 cells (Catalog #: 93-0164); CHO-K1-β-arrestin-2 MRGPRX1 cells (Catalog #: 93-0919C2); CHO-K1-β-arrestin-2 MRGPRX2 cells (Catalog #: 93-0309C2); CHO-K1-β-arrestin-2 MRGPRX4 cells (Catalog #: 93-0541C2A) form Eurofins DiscoverX, Fremont, CA, USA
GP+ envAM-12 (Catalog #: CRL-9641™); LN229 glioblastoma cells (Catalog #: CRL-2611TM) from ATCC® Manassas, Virginia, USA.
CHO-K1-β-arrestin-2-MRGPRX3; LN229-MRGPRX2 glioblastoma cells; 1321N1 Astrocytoma-MRGPRB2 cells established in the research group of the corresponding author.

Authentication

The cells were authenticated by the supplier. The receptor expression in recombinant cell lines was confirmed using immuno staining for CHO-K1-β-arrestin-2-MRGPRX3, and pharmacologically using their standard agonists for LN229-MRGPRX2 and 1321N1 Astrocytoma-MRGPRB2.

Mycoplasma contamination

Mycoplasma-free

Commonly misidentified lines
(See ICLAC register)

No commonly misidentified lines were used in this study
